# Sponge-derived matter is assimilated by coral holobionts

**DOI:** 10.1038/s42003-024-05836-z

**Published:** 2024-02-02

**Authors:** Alicia M. Reigel, Cole G. Easson, Amy Apprill, Christopher J. Freeman, Michaela M. Bartley, Cara L. Fiore

**Affiliations:** 1 Washington and Lee University, Lexington, VA USA; 2 Middle Tennessee State University, Murfreesboro, TN USA; 3 Woods Hole Oceanographic Institution, Woods Hole, RI USA; 4 College of Charleston, Charleston, SC USA; 5 Appalachian State University, Boone, NC USA

**Keywords:** Ecosystem ecology, Stable isotope analysis, Coral reefs

## Abstract

Coral reef biodiversity is maintained by a complex network of nutrient recycling among organisms. Sponges assimilate nutrients produced by other organisms like coral and algae, releasing them as particulate and dissolved matter, but to date, only a single trophic link between sponge-derived dissolved matter and a macroalgae has been identified. We sought to determine if sponge-coral nutrient exchange is reciprocal using a stable isotope ‘pulse-chase’ experiment to trace the uptake of ^13^C and ^15^N sponge-derived matter by the coral holobiont for three coral species (*Acropora cervicornis, Orbicella faveolata*, and *Eunicea flexuosa*). Coral holobionts incorporated 2.3–26.8x more ^15^N than ^13^C from sponge-derived matter and *A. cervicornis* incorporated more of both C and N than the other corals. Differential isotopic incorporation among coral species aligns with their ecophysiological characteristics (e.g., morphology, Symbiodiniaceae density). Our results elucidate a recycling pathway on coral reefs that has implications for improving coral aquaculture and management approaches.

## All

A stable isotope study elucidated a nutrient recycling pathway in which the coral holobiont incorporates nutrients from sponge-derived matter and interspecific differences in incorporation align with ecophysiological characteristics of the coral.

## Introduction

Coral reefs are characterized by high biodiversity and low nutrient availability, a paradox that is maintained by a complex network of nutrient recycling within and among organisms^1^. The transformation of major nutrients including carbon (C) and nitrogen (N) into bioavailable compounds is often attributed to planktonic communities^2^, but benthic holobionts (hosts along with their associated microbes) such as corals, algae, and sponges are also vital to reef biogeochemical cycles. For example, corals and macroalgae are net producers of dissolved organic matter (DOM) on reefs. Corals release as much as ~40% of their net photosynthetic output as mucus that dissolves and contributes to the DOM pool^1,3–6^. Marine sponges, which are prevalent filter-feeders on many coral reefs, can assimilate DOM and up to ~70–90% of the carbon uptake in some sponge species may come from DOM^7,8^. Though sponges acquire DOM from diverse sources, including algae and coral, they can selectively retain coral-derived DOM^9^ (though see also Campana et al^10^.) suggesting the coral-derived nutrients may benefit them.

Sponges and their microbial symbionts (bacteria and archaea) can process large volumes of water (as much as 35 ml min^-1^/cm^3^ tissue)^11^, assimilate dissolved (sugars, amino acids) and particulate (detritus and picoplankton)^12^ matter, and transform these nutrients into unique sponge-derived nutrients. Sponges ultimately release these nutrients as particulate detritus that may be consumed by benthic detritivores in a cycle known as the “sponge loop”^13^, and as organic and inorganic dissolved matter that leaves the sponge via ‘exhalent’ water^14–16^. The release of inorganic nitrogen in sponge exhalent water is well-documented^15,17^, but the composition of exhalent sponge-derived dissolved matter is poorly understood. However, a metabolomics study comparing ambient inhalant water to sponge exhalent water from two Caribbean sponge species found that sponge exhalent water is enriched for nucleosides and tryptophan^14^. The same study revealed that at least 71 unidentified metabolites were elevated in the exhalent relative to inhalant water^14^. Subsequent studies have found that sponge exhalent water is enriched in recalcitrant aromatic compounds^16^ and simultaneously depleted in organic nitrogen-containing compounds compared to inhalant water^18^.

The complexity of sponge-derived dissolved matter may be linked to their prokaryotic symbionts, as symbiont density and identity affects the ability of the holobiont to take up and transform nutrients (reviewed in Freeman et al^19^.), and both sponges and their microbiome can directly assimilate dissolved matter^20–22^. The specific impacts of the prokaryotic microbial community on the dissolved matter released by sponges have yet to be investigated, but sponge exhalent dissolved matter is likely composed of a mixture of sponge- and microbe-derived compounds. The quantity, as previously mentioned, and complexity of sponge-derived dissolved matter suggests that it may be utilized by a wide variety of surrounding organisms on the reef. Indeed, a single study using stable isotope tracers found a beneficial transfer of sponge-produced dissolved matter to macroalgae^23^, but to date, no other trophic links fueled specifically by sponge-derived dissolved matter have been uncovered.

Corals thrive on oligotrophic reefs in part because of a mutualism with endosymbiotic micro-algae belonging to the family Symbiodiniaceae^24^. The importance of resource sharing and recycling, particularly of carbon- and nitrogen-containing nutrients, between the holobiont fractions (i.e., host and micro-algae) has been demonstrated for many coral species^25,26^. Symbiodiniaceae harness sunlight to produce carbon-rich photosynthates in excess of their own needs, translocating as much as 95% of them to their coral host^27,28^. Despite their high carbon content, photosynthates are nitrogen and phosphorus poor, meaning that corals must seek these vital nutrients elsewhere^27,29^. Corals thus rely on heterotrophic feeding to acquire nitrogen and other limiting nutrients (e.g., phosphorous, iron)^30^. Healthy corals will actively feed on zooplankton and dissolved and particulate matter, obtaining an estimated 15–35% of their carbon and >70% of their nitrogen demand^30,31^ from these external sources. There is also evidence of nitrogenous compound exchange between coral and Symbiodiniaceae^25,26^, and this internal nitrogen flux is thought to account for the majority (90–98%) of the nitrogen needs of the micro-algae^32,33^. Although the contribution of heterotrophic feeding on zooplankton and particulate matter by the coral holobiont is well-understood, there is limited information about the sources of dissolved matter that may be utilized by the distinct fractions.

Given the importance of nutrient cycling on coral reefs and our knowledge of the contribution of sponges to dissolved matter cycling, we hypothesized that the exchange of nutrients between the coral and sponge holobionts is reciprocal, with corals assimilating sponge-derived dissolved matter into their tissues. Here, we describe the first test of this hypothesis using two Caribbean scleractinian corals commonly used in efforts to restore Florida and Caribbean reefs, *Acropora cervicornis* and *Orbicella faveolata*, considered ‘critically endangered’^34^ and ‘endangered,’^35^ respectively, by the IUCN, as well as the prevalent octocoral *Eunicea flexuosa*. To address this question, we used a stable-isotope ‘pulse-chase’ experiment in which compounds containing heavy isotopes of C and N (^13^C and ^15^N) were provided to sponges during a ‘pulse.’ Following the ‘pulse,’ sponges were placed into a tank with coral fragments in a 6-hr ‘chase’ where enriched sponge-derived dissolved matter could be taken up by the corals. Sponge-derived nutrients assimilated by the coral and Symbiodiniaceae were then traced by looking at changes in the concentration of heavy isotopes in their tissues.

We further predicted that any uptake of sponge-derived dissolved matter by corals would be differential (1) across nutrient types (i.e., C and N) with a higher uptake of nitrogenous dissolved matter due to its limited concentration on reefs, (2) between holobiont fractions (A: coral including the prokaryotic community, B: Symbiodiniaceae) due to their symbiotic relationship and distinct metabolic pathways, and (3) across coral species given their potential for differential reliance on heterotrophic nutrient acquisition based on structure, morphology, and the composition of their symbiont (Symbiodiniaceae and microbes) community^30,36^.

## Results

### Sponge-derived dissolved matter labeling

To trace the uptake of sponge-derived dissolved matter by the coral holobiont, we first exposed six species of Caribbean sponges (*Niphates digitalis*, *Verongula rigida*, *Aplysina fulva*, *Aplysina cauliformis*, *Iotrochota birotulata*, *Callyspongia aculeata*) to isotopically labeled inorganic sodium bicarbonate (NaH^13^CO_3_), sodium nitrate (Na^15^NO_3_), and ammonium chloride (^15^NH_4_Cl) in a 3 hr ‘pulse’. Following the ‘pulse’ the sponges were enriched in both ^13^C and ^15^N and this isotope signal decreased during the ‘chase’ as sponges released the isotopes in enriched sponge-derived matter. Sponges were thoroughly rinsed for 1 h in flowing seawater to remove any excess label before placing them in the ‘chase’ tanks with coral fragments (*n* = 3 each from *A. cervicornis*, *O. faveolata* and *E. flexuosa*). In theory, assimilated labeled compounds would be transformed by the sponge and/or microbial symbionts for use in growth, metabolite production, and respiration. The subsequent release of the label during the ‘chase’ could occur by (1) excretion of dissolved metabolites, (2) dissolved inorganic compounds from respiration, and/or (3) cell turnover. We expect most labeled sponge-derived matter to be dissolved matter but acknowledge that we do not know the proportion of dissolve organic nutrients vs inorganic nutrients (e.g., ammonia) containing the label. Regarding cell turnover, there was no visible detritus in the experimental tanks suggesting that detritus production was low; additional detail can be found in the Methods. Additionally, there may be uptake and subsequent release of labeled nutrients by the corals, but the labeled nutrients are ultimately sponge derived. For simplicity, we will refer to all labeled sponge-derived nutrients as dissolved matter (DM), or specifically, carbon-containing or nitrogenous dissolved matter (CDM and NDM, respectively).

**Fig. 1:**
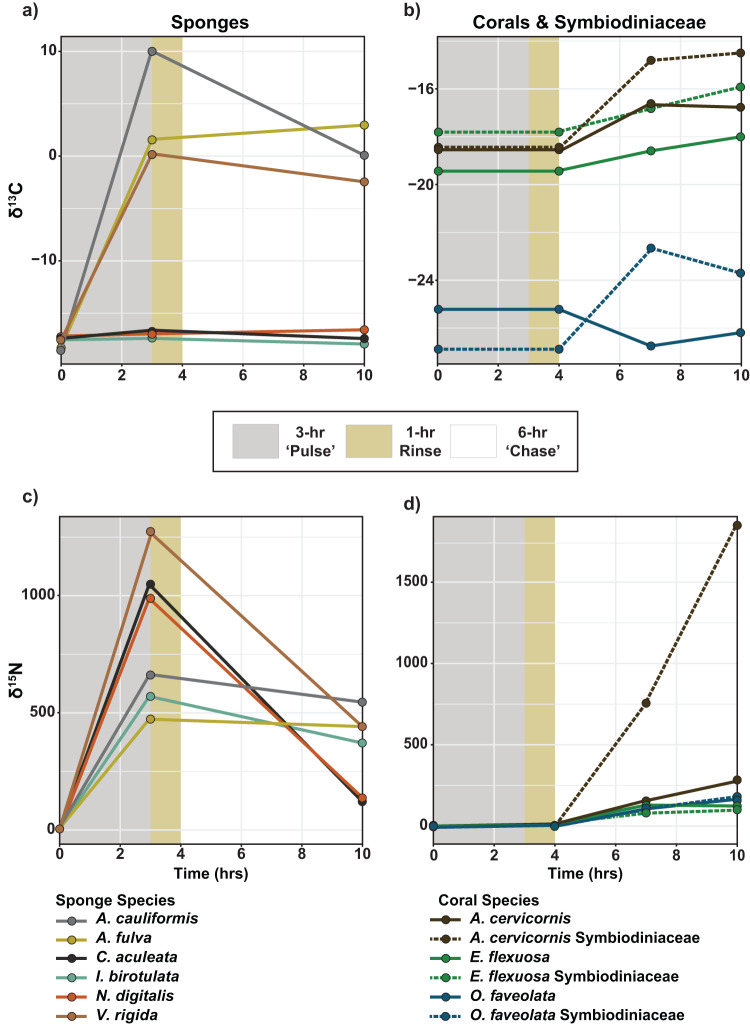
The δ ^13^C and δ ^15^N (^0^/_00_) values in sponges, corals, and Symbiodiniaceae during the 10-hr “pulse-chase” experiment. The change in *δ* ^13^C (**a**) for sponges and (**b**) coral and Symbiodiniaceae, and the change in *δ* ^15^N for (**c**) sponges and (**d**) coral and Symbiodiniaceae. Sponge species include *Aplysina cauliformis* (gray line), *Aplysina fulva* (yellow line), *Callyspongia aculeata* (black line), *Iotrochota birotulata* (light green), *Niphates digitalis* (orange line), and *Verongula rigida* (light brown line). Coral (solid line) and Symbiodiniaceae (dashed line) are from three species *Acropora cervicornis* (dark brown), *Eunicea flexuosa* (green), and *Orbicella faveolata* (blue). The 10 h experiment is divided into three sections: 3 h ‘pulse’ (gray shaded area), 1 h rinse (gold shaded area), and 6-h ‘chase’ (white shaded area). Hours 0, 7 and 10 correspond to T_0_, T_3_ and T_6_ sampling points, respectively, and hour 3 corresponds to the ‘pulse’ sampling point for sponges only. Corals and Symbiodiniaceae were only exposed to enriched sponges during the ‘chase.’ Sample size information can be found in Supplementary Table S1.

The sponge, *A. cauliformis* showed the largest increase in *δ* ^13^C during the ‘pulse’, followed by *A. fulva* and *V. rigida* (+28.6‰, +19.9‰, and +17.7‰ compared to their own natural abundance values, respectively; Fig. 1a and Supplementary Table S2). The other three sponge species had <+1 ^0^/_00_ change in *δ* ^13^C following the pulse (Fig. 1Aa). All six sponge species had increased *δ* ^15^N following the ‘pulse’, but the greatest increases were in *V. rigida*, *C. aculeata* and *N. digitalis* (+1268.8‰, +1044.7‰, and +983.3‰ compared to natural abundance values, respectively; Fig. 1b and Supplementary Table S2). However, all six species had a reduction in ^15^N (from -198.7 to–927.4‰) during the ‘chase’ (Fig. 1b). Due to low sample sizes, statistical analysis was not performed on sponge samples. Lastly, comparing bulk nutrients in the ‘pulse’ and ‘chase’ seawater revealed no difference in DOC concentrations (Student’s *t*-test; *t*_2.28_ = −0.343, *p* = 0.791; Supplementary Fig. S3). In contrast, TN values were significantly higher in the ‘pulse’ than the ‘chase’ (Kruskal-Wallis χ^2^_1_ = 3.857, *p* = 0.005; Supplementary Fig. S3).

### Assimilation of sponge-derived DM into coral and symbiodiniaceae

**Fig. 2:**
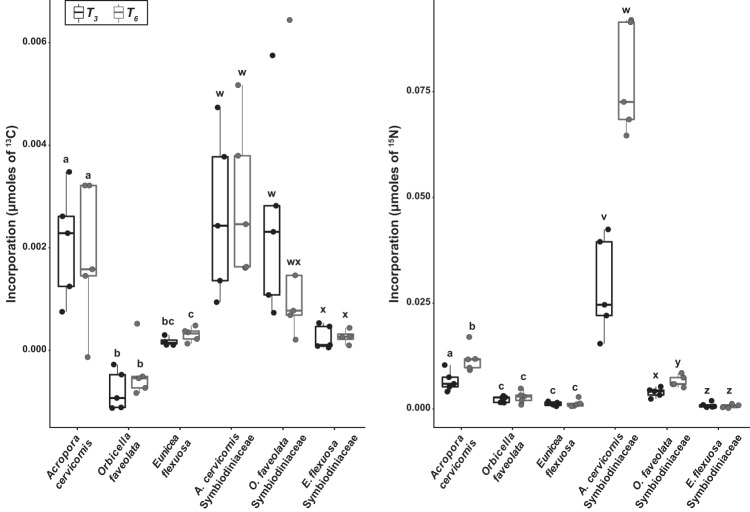
Incorporation of labeled isotopes from sponge-derived nutrients by the coral holobiont. Incorporation (µmol) of (**a**) ^13^C, and (**b**) ^15^N from sponge-derived compounds by coral and associated Symbiodiniaceae for three species (*Acropora cervicornis*, *Orbicella faveolata* and *Eunicea flexuosa*) at both T_3_ (black boxes) and T_6_ (gray boxes) ‘chase’ sampling points. Letters within each figure panel denote significant differences among species and sampling points, based on pairwise PERMANOVA tests with Bonferroni corrections, between coral (a–c) and Symbiodiniaceae (v–z) tissues. Letters do not correspond to any differences between fractions. The lower and upper hinges of each box represent the first and third quartiles. The lower and upper whiskers extend to either the largest or smallest value that is no more than 1.5 x the interquartile range (i.e., distance between the first and third quartiles). Outliers are plotted as individual points. Sample size information can be found in Supplementary Table S1.

**Fig. 3:**
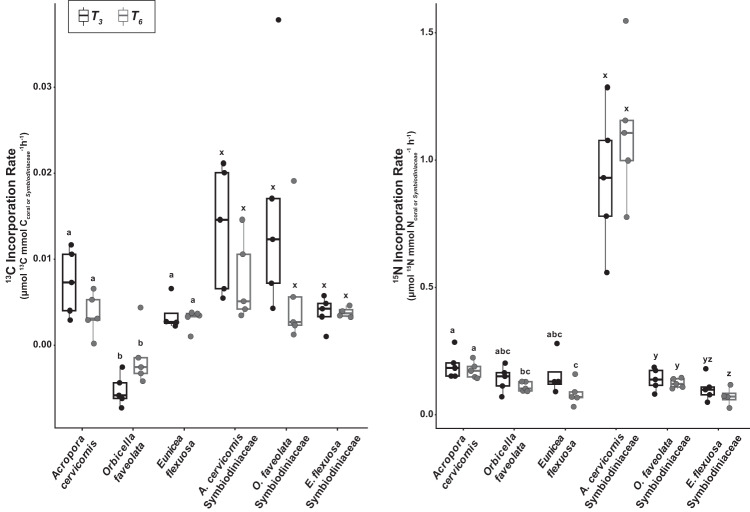
Incorporation rates of labeled isotopes from sponge-derived dissolved matter by the coral holobiont. Incorporation rates of (**a**) ^13^C, and (**b**) ^15^N by coral and Symbiodiniaceae from three species (*Acropora cervicornis*, *Orbicella faveolata* and *Eunicea flexuosa*) are represented as the µmol of ^13^C or ^15^N per mmol of C or N in the initial sample per hour of exposure to heavy isotope (i.e., per hours in the ‘chase’). The per hour rate for the first 3 h of the ‘chase’ (T_3_) is denoted by black boxes, and the per hour rate across the entire 6 h ‘chase’ (T_6_) is denoted gray boxes. Letters within each figure panel denote significant differences among species and sampling points, based on pairwise PERMANOVA tests with Bonferroni corrections, between coral (a–c) and Symbiodiniaceae (x–z) tissues. Letters do not correspond to any differences between fractions. The lower and upper hinges of each box represent the first and third quartiles. The lower and upper whiskers extend to either the largest or smallest value that is no more than 1.5 x the interquartile range (i.e., distance between the first and third quartiles). Outliers are plotted as individual points. Sample size information can be found in Supplementary Table S1.

During the 6-hr ‘chase,’ ^13^C and ^15^N increased in corals and their Symbiodiniaceae that were exposed to enriched sponges (Fig. 1c, d). Symbiodiniaceae incorporated significantly more ^13^C and ^15^N than their coral counterparts. However, measurements of isotope incorporation above background values also takes into account the natural micromole amount of N or C content of the sample. Isotope incorporation rate, which normalizes incorporation to the time the sample was exposed to the enriched DM, also varied within fractions by coral species and across sampling time points (T_3_ and T_6_: the midpoint and end of the 6-h ‘chase’, respectively; Figs. 2, 3 and Supplementary Table S3). Significant differences are reported as *p*-values in text, and detailed PERMANOVA test results can be found in Supplementary Table S4 with corresponding post-hoc pairwise tests found in the Supplementary Information (pgs. 18–21).

### Incorporation of ^13^C from sponge-derived DM

Following the 6 h ‘chase,’ the holobionts exposed to the enriched sponge-derived DM had significant changes in *δ*^13^C (PERMANOVA, *p* = 0.004). All tissues, except for *O. faveolata*, had a net positive change in *δ*^13^C, but the Symbiodiniaceae fraction had a greater average net increase (+3.0‰) than the coral fraction (+0.7‰; PERMANOVA, *p* = 0.001). Within each fraction, the change in *δ*^13^C differed across host species (PERMANOVA, *p* = 0.001). *A. cervicornis* Symbiodiniaceae had the greatest increase (+4.0‰), followed by Symbiodiniaceae from *O. faveolata* and *E. flexuosa* (+3.2‰ and +1.8‰, respectively; Fig. 1c). *O. faveolata* had an overall decrease in *δ*^13^C of −1.0‰ throughout the ‘chase’ indicating that ^13^C was lost, rather than gained by this coral (Fig. 1c). The *δ*^13^C values for control samples, which were not exposed to enriched sponges, were significantly different between fractions and among species (PERMANOVA, both *p* = 0.001). Additionally for control samples held in aquaria without enriched sponges there was no significant change in *δ*^13^C following the ‘chase’ (for either fraction (PERMANOVAs, all *p* > 0.05)).

^13^C incorporation ranged from 0 to 0.0029 µmol (Supplementary Table S3) and varied significantly between enriched coral and Symbiodiniaceae fractions (PERMANOVA, *p* = 0.007; Fig. 2a). Within the coral host fraction, there was differential ^13^C incorporation among the species (PERMANOVA, *p* = 0.001), with *A. cervicornis* incorporating significantly more ^13^C than both *E. flexuosa* and *O. faveolata* (pairwise PERMANOVAs, both *p* = 0.001). Similarly, *E. flexuosa* had higher ^13^C incorporation than *O. faveolata* (pairwise PERMANOVA, *p* = 0.002). The Symbiodiniaceae fraction from different host species also had differential ^13^C incorporation (PERMANOVA, *p* = 0.011) with patterns mirroring those seen in the δ^13^C data. Incorporation of ^13^C from sponge-derived DM by the coral holobiont appears to reach its limit within the first half of the 6-hr ‘chase,’ as neither coral (PERMANOVA, *p* = 0.756) nor Symbiodiniaceae (PERMANOVA, *p* = 0.859) exhibited a significant change in ^13^C incorporation between T_3_ and T_6_ sampling points.

^13^C incorporation rates, which normalize incorporation to the time interval of exposure to enriched sponge-derived DM in the ‘chase’, were also significantly different among fractions (PERMANOVA, *p* = 0.001) with Symbiodiniaceae incorporating ^13^C at a ~6x higher rate than their coral hosts (Fig. 3a). Within the coral fraction, the ^13^C incorporation rates match the patterns of ^13^C incorporation, showing no difference in rate between the first 3 h interval (i.e., T_0_-T_3_) and the entire 6-h interval (i.e., T_0_-T_6_) of the ‘chase’ (PERMANOVA, *p* = 0.908; Fig. 3a). However, there were differences among the species (PERMANOVA, *p* = 0.001) with *A. cervicornis* and *E. flexuosa* exhibiting a significantly higher rate than *O. faveolata* (pairwise PERMANOVAs, both *p* = 0.001; Fig. 3a). *O. faveolata*’s incorporation rate for ^13^C was negative, matching the previously reported decrease in *δ*^13^C during the ‘chase; and indicating a lack of uptake of sponge-derived CDM by this coral. Among the Symbiodiniaceae fraction, neither host species nor time interval of exposure to sponge-derived DM impacted ^13^C incorporation rates (PERMANOVA tests, *p* > 0.05; Fig. 3a).

### Incorporation of ^15^N from sponge-derived nitrogenous DM

In contrast to *δ*^13^C, the *δ*^15^N values were much higher, and all positive, for both coral and Symbiodiniaceae during the ‘chase’ (Supplementary Table S2). *A. cervicornis* Symbiodiniaceae exhibited a significantly greater net increase in δ^15^N (+1826.4‰) than all other coral and Symbiodiniaceae (PERMANOVA, *p* = 0.001; pairwise PERMANOVAs, all *p* > 0.05). The second greatest increase in *δ*^15^N was in *A. cervicornis* (+268.5‰), followed by *O. faveolata* Symbiodiniaceae (183.2‰; Fig. 1d). *E. flexuosa* Symbiodiniaceae had the smallest overall increase of only +21.0‰ (Fig. 1d). In the control samples, the *δ*^15^N values differed between the fractions, but not among the species (PERMANOVAs, *p* = 0.049 and *p* = 0.597, respectively). *δ*^15^N did increase in control *A. cervicornis* (PERMANOVA, p = 0.011;) and *E. flexuosa* (PERMANOVA, *p* = 0.047) from T_0_ to T_3_ (pairwise PERMANOVAs, both *p* = 0.041), but for both corals this difference disappeared between T_3_ and T_6_ (pairwise PERMANOVA tests, all *p* > 0.05). There were no differences in *δ*^15^N across the ‘chase’ for any of the other control holobiont tissues (PERMANOVAs, all *p* > 0.05).

^15^N incorporation for coral and Symbiodiniaceae during the ‘chase’ reflected the same pattern as the *δ* ^15^N data (Fig. 2b and Supplementary Table S3). On average, the Symbiodiniaceae fraction incorporated significantly more ^15^N (0.02 µmol) than their coral counterparts (0.0004 µmol; PERMANOVA, *p* = 0.013). Among the coral (PERMANOVA, *p* = 0.001) and Symbiodiniaceae (PERMANOVA, *p* = 0.001) fractions, incorporation differed between the species, and *A. cervicornis* host and micro-algae incorporated more ^15^N than their fraction-specific counterparts from the other two species (pairwise PERMANOVAs, all *p* = 0.001; Fig. 2b).

Incorporation rates of ^15^N from sponge-derived NDM were ~10 fold greater than for CDM (Fig. 3a and Supplementary Table S3). The ^15^N incorporation rate of the Symbiodiniaceae fraction was ~4x as high as that of the coral fraction (PERMANOVA, *p* = 0.008), and the rates also differed among holobiont species within fractions (PERMANOVA, Coral fraction: *p* = 0.006, Symbiodiniaceae fraction: *p* = 0.001). Similar to ^15^C incorporation rate, there was no difference in rates between time intervals (T_0_-T_3_ and T_0_-T_6_) of exposure to enriched sponge-derived DM for either fraction (PERMANOVA, both *p* > 0.05). Overall, *A. cervicornis* Symbiodiniaceae had the highest ^15^N incorporation rate of all coral and Symbiodiniaceae at both time intervals (T_0_-T_3_ and T_0_-T_6_; Fig. 3b).

### Coral Holobiont traits: carbon and nitrogen trends in corals and zooxanthellae abundances

To gain a better understanding of the potential for nitrogen limitation in the coral holobiont, we examined the molar C:N ratios of the T_0_ samples. The average molar C:N ratio of 5.94 for the Symbiodiniaceae fraction was significantly lower than the average ratio of 6.36 of the coral fraction (PERMANOVA, *p* = 0.016). Within the fractions there were also differences between species (PERMANOVA, *p* = 0.001). *A. cervicornis* and *O. faveolata* had significantly higher C:N ratios than the octocoral, *E. flexuosa* (pairwise PERMANOVAs, all *p* = 0.001; Supplementary Fig. S4). Additionally, *A. cervicornis O. faveolata* host tissue had a significantly higher C:N ratio than their associated Symbiodiniaceae (Student’s *t*-tests, t_5.71_ = 3.51, *p* = 0.0134 and *t*_3.14_ = 5.65, *p* = 0.010, respectively; Supplementary Fig. S4), but the opposite was true for *E. flexuosa* whose Symbiodiniaceae had a higher C:N ratio (Student’s *t*-test, *t*_6.75_ = −2.733, *p* = 0.03; Supplementary Fig. S4). Within the coral fraction, *E. flexuosa* had the lowest C:N ratio, while *O. faveolata* Symbiodiniaceae had the lowest ratio among the Symbiodiniaceae (Supplementary Fig. S4).

Symbiodiniaceae density (cells/cm^2^) differed across the three coral species, but was not impacted by treatment (i.e., exposure to enriched sponge-derived DM; PERMANOVA, Species: *p* = 0.001, Treatment: *p* = 0.133). *O. faveolata* had a denser Symbiodiniaceae community than *A. cervicornis*, and both scleractinians had denser Symbiodiniaceae communities than the octocoral, *E. flexuosa* (pairwise PERMANOVAs, all *p* = 0.001; Supplementary Fig. S5). Neither scleractinian displayed a change in algal density among time points (PERMANOVAs, all *p* > 0.05; Supplementary Fig. S5), but algal density in *E. flexuosa* was variable across time (PERMANOVA, *p* = 0.006) with T_3_ samples slightly less dense than T_6_ samples (pairwise PERMANOVA, *p* = 0.001; Supplementary Fig. S5).

## Discussion

We enriched emergent Caribbean sponges with ^13^C and ^15^N and traced, for the first time, these compounds into the coral holobiont, elucidating a novel, to the best of our knowledge, energetic link between corals and sponges. The results presented here support each of our initial hypotheses: (1) that corals assimilate nutrients released by sponges, and that the assimilation of nutrients would vary by, (2) nutrient type with higher assimilation of ^15^N-containing compounds relative to those containing ^13^C, (3) holobiont fraction (i.e., host and Symbiodiniaceae), and (4) coral species. Here, we discuss the evidence for a recycling pathway from sponges to corals on shallow Caribbean reefs.

The enrichment and subsequent release of labeled dissolved nutrients was different among the six sponge species. Sponge incorporation of inorganic ^15^N during the ‘pulse,’ was higher (1.9-300x more) than for ^13^C. The relatively low incorporation of ^13^C overall in sponges may be due to low irradiance during the experiment (ranged from 150–300 PAR (μMol photons/m^2^/s))^37^, and/or our use of a single, non-complex isotope source (inorganic bicarbonate) during the ‘pulse’, as bicarbonate tends to target autotrophic metabolic pathways and its uptake may be hindered by low light levels. Three of the six sponge species incorporated only negligible amounts of the ^13^C isotope, but those that incorporated the most ^13^C from the labeled bicarbonate were the Cyanobacteria-containing species *V. rigida, A. cauliformis*, and *A. fulva*^38^. In comparison, during a 6-hr in-situ study of the uptake of complex enriched diatom-derived DOM, sponges had a 2x greater increase in *δ*^13^C than those in our study^13^. In contrast, nitrate and ammonium are readily taken up by autotrophic and heterotrophic microbes and the sponge hosts (e.g., Fiore et al^17^.). Thus, the clear incorporation of the ^15^N inorganic nitrogen was expected, and all sponge species did show enrichment. Release of ^13^C and to a greater extent, ^15^N by the sponges was observed via the decrease in isotopic signal in the sponges over time during the ‘chase’ period. We expect that this was largely released as dissolved organic matter as sponges have been documented to alter the seawater DOM profile as they filter;^14,16^ however, sponges may have also released the ^13^C and/or ^15^N as particulate matter and as inorganic nitrogen.

The labeled sponge-derived nutrients were assimilated by corals providing the first evidence of the heterotrophic acquisition of sponge-derived DM by the coral holobiont. It is possible that there was additional cycling of the sponge-derived matter by corals. For instance, some corals could have rapidly assimilated sponge derived compounds and released these back into the tank via respiration or turnover. Despite this, the ultimate source of the heavy isotope in all samples was from sponges. It is generally accepted that coral health is dependent on photosynthates (i.e., carbohydrates) provided by their symbiotic Symbiodiniaceae, but further work has uncovered the importance of heterotrophic feeding (on plankton, POM and DOM) by corals to obtain limiting nutrients not provided by photosynthate, such as nitrogen^30^. We observed incorporation of sponge-derived DM by corals, but the amount of incorporation differed across the examined elements. Sponge-derived CDM was incorporated by two of the three coral hosts, *A. cervicornis* and *E. flexuosa*, and the Symbiodiniaceae of all three coral species, while both the host and Symbiodiniaceae fractions of all three species incorporated sponge-derived NDM. Differential incorporation of ^13^C and ^15^N among holobiont species may be related to host features including morphology, reliance on heterotrophic feeding, and the identity of both their Symbiodiniaceae and prokaryotic symbionts.

*A. cervicornis* host tissue had the greatest increase in *δ* ^13^C across the 6 h ‘chase’, and their quick incorporation of the enriched carbon within the first half of the ‘chase’, suggests the potential for direct uptake by the host fraction. However, translocation of fixed carbon from Symbiodiniaceae can occur as soon as 15 min^39^, supporting translocation of carbon acquired by the Symbiodiniaceae to *A. cervicornis* host tissue. Interestingly, both the Symbiodiniaceae fraction and host fraction increased in ^13^C at T_3_ relative to the control samples, but only the Symbiodiniaceae continued to increase over the next 3 h. This pattern does not support continued translocation of labeled carbon acquired by Symbiodiniaceae to *A. cervicornis*, where we would expect a decrease in ^13^C in Symbiodiniaceae and an increase in the coral host. Heterotrophic consumption of DOM by corals is well-documented, although there is limited information on the extent of reliance on DOM among coral species (reviewed in Houlbrèque & Ferrier-Pagès^30^). However, one study observed the differential use of heterotrophy across Hawaiian coral species^40^ suggesting unique nutrient strategies for different species. Our data suggest that *A. cervicornis* in the Florida Keys may be mixotrophic and incorporates some exogenous carbon compounds, specifically, sponge-derived exogenous carbon, in addition to Symbiodiniaceae-derived photosynthates.

In contrast, the other scleractinian *O. faveolata* assimilated little to none of the sponge-derived ^13^C, with a net negative ^13^C flux. Limited ^13^C incorporation may indicate a heavy reliance on photosynthates from their abundant Symbiodiniaceae community rather than heterotrophy, which was found to account for <10% of their fixed carbon budget^41^. Because an organism’s carbon isotope composition is similar to that of their food source, the low C:N value of the initial *O. faveolata* fragments, matching that of its micro-algal symbionts, suggests that the boulder coral relies heavily on translocated nutrients. In further support of carbon translocation from *O. faveolata* symbiont to host, ^13^C increased in the host fraction between T_3_ and T_6_ and decreased in the corresponding Symbiodiniaceae fraction.

*E. flexuosa* incorporated a moderate amount of ^13^C from sponge-derived DM during the ‘chase’ relative to the other two hosts. *E. flexuosa* also had the lowest density of algal cells, which likely necessitates some heterotrophic nutrient acquisition, either of dissolved or particulate matter, by the host. Additionally, both the *E. flexuosa* host and the Symbiodiniaceae fraction increased in ^13^C continuously, albeit moderately, throughout the ‘chase’ supporting incorporation of ^13^C DM but no or minimal translocation between algal symbiont and host. Heterotrophy in *E. flexuosa* was also supported by an initial *δ*^13^C value in the coral tissue that was lower than that of its Symbiodiniaceae symbionts, a common signature of heterotrophy^42^. Octocoral morphology is well-suited to the uptake of particulate organic matter because of pinnules on each tentacle that can act as fine sieves for nutrients and prey^42^. Further, the incorporation of ^15^N in *E. flexuosa* was quite low compared to the scleractinian corals, which could result from the consumption of low amounts of sponge-derived detritus and/or a limited ability to assimilate dissolved nitrogenous nutrients^43,44^.

Coral hosts do rely on the photosynthates produced by their Symbiodiniaceae for carbon, but photosynthates are typically considered “junk food” because of the high C:N ratio^29^. Therefore, corals must seek out additional nitrogen sources to sustain their growth. In our study, all three coral species did incorporate ^15^N, but there were differences among species that are likely attributed to their own ability to acquire sponge-derived NDM via heterotrophy and/or the nitrogen-fixing abilities of their prokaryotic symbionts.

The C:N values of, *A. cervicornis* and *O. faveolata* were higher than that of *E. flexuosa*, which supports a stronger need for nitrogen in the scleractinians relative to the octocoral. Indeed, *A. cervicornis* and *O. faveolata* incorporated more sponge-derived ^15^N, while *E. flexuosa* had limited assimilation of sponge-derived ^15^N-containing compounds. Generally, octocorals display less nitrogen depletion, even at shallow depths, than their scleractinian counterparts^43,45^, likely due to heterotrophic feeding on both zooplankton and POM^46^. The sponge-derived compounds in this study are predominantly dissolved rather than particulate and may not be as readily acquired by the octocoral. The apparent differential reliance on heterotrophy among our three coral species is reinforced by their concentrations of Symbiodiniaceae cells. *E. flexuosa* fragments have one to two orders of magnitude lower density of Symbiodiniaceae (at T_0_) than the scleractinians that are more reliant on microalgal-derived photosynthates.

Outside of heterotrophic acquisition, corals can also obtain nitrogen from their symbiotic nitrogen-fixing prokaryotic microbes. At least one of the stony corals in our experiment. *O. faveolata*, harbors a diverse symbiotic diazotrophic community capable of each step of the nitrogen cycle, including nitrogen-fixation^47^, which may explain the lower incorporation of ^15^N in this species compared to the other scleractinian coral host. Nitrogen cycling in *E. flexuosa* is not well characterized, but studies have documented a positive correlation between nitrogen assimilation and increased nutrient availability^48,49^. The *E. flexuosa* prokaryotic microbiome has a relatively high abundance of the common coral-associated bacteria *Endozoicomonas*^48,50^ that is capable of reducing nitrate to bioavailable ammonium and is suspected to play an important role in nitrogen acquisition for its coral host^51,52^. More broadly, soft corals are often found in higher abundance than scleractinians on shallow, low-quality reefs^53,54^. On these low-quality reefs, heterotrophy would benefit octocorals because the turbid, low light waters limit the ability of photo-diazotrophic symbionts to fix nitrogen for their hosts^43^ and at least one study indicated limited support for an active diazotrophic community in octocorals^44^. Similarly, the prokaryotic microbiome of *A. cervicornis* is not well-characterized in terms of function, but profiling of this species in the Caribbean, including in the lower Florida Keys reef tract, does not indicate a predominance of nitrogen-cycling taxa^55^, which may explain their increased uptake of ^15^N from sponge-derived DM compared to the other corals. However, prokaryotic symbionts capable of nitrogen fixation were present and documented to increase with depth in Puerto Rican *A. cervicornis* individuals^56^, suggesting that there may be location- and depth-specific differences in nitrogen cycling for this coral holobiont.

Despite initial variation in C:N of the corals, and differences in both morphology and symbiont communities, the host fraction of all three coral species did assimilate sponge-derived DM. Previous work in scleractinian corals suggests translocation of N from Symbiodiniaceae to host begins around 3 h^39^, thus the increase in ^15^N in host tissue between T_0_ to T_3_ in our study suggests some direct acquisition of N by the coral host. However, there may be a higher need for nitrogen compounds by *A. cervicornis* host tissue in particular, as this species increased the most in ^15^N during the 6-hr ‘chase’. While our time series data gives evidence for uptake by both fractions, additional targeted experiments would be needed to parse direct uptake by the host versus translocation from their Symbiodiniaceae. For *A. cervicornis*, the results presented here supporting direct N acquisition from sponge-derived matter by the coral host have management implications as *A. cervicornis* is favored in nurseries as a fast-growing species that could propagate successfully on reefs^57^. The success of the fragments is tied to a mix of genotype and environmental factors^58^ and our results suggest that nitrogen availability may be an important environmental factor and that sponges may be able to buffer against nitrogen limitation in *A. cervicornis*.

We also examined the uptake of both labeled isotopes, ^13^C and ^15^N, by the Symbiodiniaceae fraction of the coral holobiont. We observed incorporation of ^13^C from sponge-derived CDM by Symbiodiniaceae belonging to all three coral hosts. Scleractinian-associated Symbiodiniaceae had higher ^13^C incorporation than their hosts, supporting similar results from another coral ‘pulse-chase’ experiment^59^. *E. flexuosa* Symbiodiniaceae incorporated the same amount of ^13^C as their host, which may be a result of the host’s ability to acquire their own carbon via heterotrophy. Interestingly, *O. faveolata* Symbiodiniaceae incorporated >3x as much ^13^C from sponged-derived CDM as their host. However, during the last half of that chase the O. *faveolata* Symbiodiniaceae had a net loss of ^13^C that corresponded to a net gain in the *O. faveolata*, suggesting carbon translocation from Symbiodiniaceae to host^27,28,45^.

Differential uptake of sponge-derived CDM among the Symbiodiniaceae from different coral species correlated positively with their densities. Typically, Symbiodiniaceae rely on their hosts to supply inorganic carbon in the form of CO_2_^45^, a limiting factor for photosynthesis^60^. However, CO_2_ depletion is common in corals with high densities of Symbiodiniaceae^61^, like our scleractinians. When the coral is CO_2_ depleted (i.e., when photosynthesis exceeds respiration rates) the Symbiodiniaceae may take up ^13^C directly, usually in the form of bicarbonate^62,63^. It is not clear from our study if the sponge is producing labeled bicarbonate (e.g., labeled CO_2_ from sponge respiration that forms bicarbonate in seawater), but if so, this may explain the higher incorporation of ^13^C in the denser scleractinian Symbiodiniaceae populations, as compared to the octocoral.

The uptake of ^15^N from sponge-derived NDM was very high among the Symbiodiniaceae. This result was expected as previous work acknowledged that Symbiodiniaceae are responsible for most of the DIN uptake in the symbiosis^64^, and nitrogen limitation is considered the primary mechanism for the coral holobiont to control the growth of Symbiodiniaceae^52,65^. Turnover of nitrogen within Symbiodiniaceae is often high^24^, but varies across host species highlighting both the importance of this resource in algal growth and different nitrogen budgets across Symbiodiniaceae housed by different hosts.

Symbiodiniaceae from *A. cervicornis* readily took up ^15^N, and following the ‘chase’ had >10x *δ*^15^N than the Symbiodiniaceae from both other corals. The *A. cervicornis* Symbiodiniaceae were also the only holobiont tissue with a higher ^15^N incorporation rate in the second half of the ‘chase’ supporting a strong need for acquisition of exogenous nitrogen. Differential uptake of ^15^N among the Symbiodiniaceae from different hosts may be, at least partially, explained by their own physiological capabilities. Symbiodiniaceae are highly diverse, encompassing seven genetically and physiologically unique genera (formerly clades A–G)^24^. Natural values of C:N vary among the genera^66^ and some have an enhanced ability to acquire nitrogen, which may benefit their host via nutrient translocation and lead to enhanced coral growth rates^67^. *A. cervicornis*, is a fast-growing coral (~71 mm yr^-1^)^68^, and thus may require Symbiodiniaceae that are efficient at nitrogen acquisition. We did not genotype the Symbiodiniaceae in our study, but differences in their composition among the holobiont species could explain some of the striking differences we observed in their ^15^N incorporation.

Over the last century coral reefs have rapidly deteriorated due to multiple stressors associated with human activity^69^. As a result, coral cover has decreased and macroalgae cover, and in some cases sponge cover, has increased, likely due to their higher tolerances for eutrophication and sedimentation, among other factors^70^. As the composition of the benthic community shifts, it is vital that we gain a better understanding of the potential impacts these shifts will have on reef-wide biogeochemical cycles. For example, increased macroalgae cover on reefs, and corresponding increases in labile algae-derived exudates, promotes “microbialization” resulting in hypoxic conditions and increased CO_2_ further degrading reef quality^4,71^. Similarly, the release of sponge-derived nutrients has been suggested to induce a positive feedback loop that may promote sponge and macroalgae growth leading to further declines in coral cover^9,72^. However, this positive feedback loop between sponges and macroalgae growth has only been tested in a limited capacity, where the first assimilation of sponge-derived nitrogen by the green alga *Microdictyon marinum* was quantified using the sponge *A. cauliformis*^23^. To our knowledge there is not any other documentation of the effect of sponge-derived dissolved material on macroalgae or other coral reef species.

**Fig. 4:**
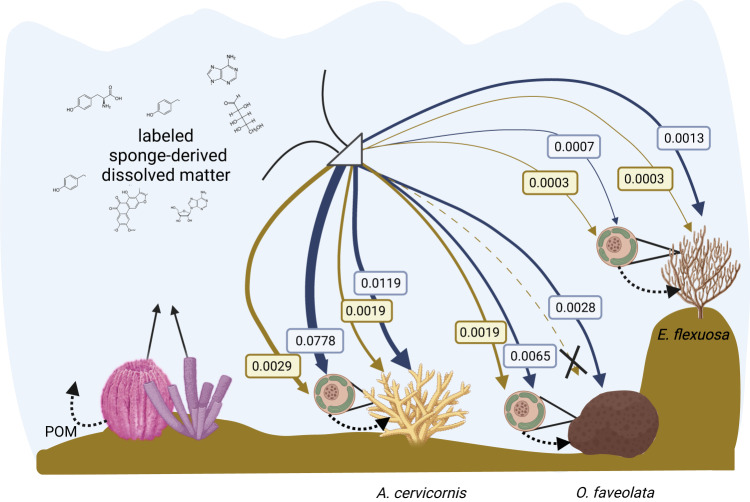
Conceptualization of the incorporation of sponge-derived compounds by three coral host species and their associated Symbiodiniaceae. Depiction of the incorporation of sponge-derived ^13^C (brown arrows) and ^15^N (blue arrows) for each coral fraction (host and Symbiodiniaceae). Values shown with each arrow are µmoles of carbon (^13^C) or nitrogen (^15^N) in the coral or Symbiodiniaceae at the end of the 6 h ‘chase.’ Dotted black arrows denote potential nutrient exchanges between host and symbiont within the coral holobiont, and the potential release of enriched particulate organic matter (POM) by the sponge community. Created with Biorender.com.

Here we provide the first evidence for assimilation of sponge-derived DM by corals, elucidating a trophic link between sponges and corals in which sponges provide carbon and importantly, nitrogen to corals and their Symbiodiniaceae (Fig. 4). Determining the specific compounds and forms of acquired sponge-derived matter (i.e., dissolved organic and/or inorganic) and the extent to which labeled particulate matter was produced by sponges and consumed by corals would be valuable to address in future studies. Additionally, determining the extent to which this sponge to coral trophic link occurs in nature and under what circumstances it occurs, is now warranted in future work. This work builds on our nascent understanding of the release of DOM by sponges^14,16^ and the ‘sponge loop’ where sponges consume DOM and release particulate matter that is assimilated by the benthic invertebrate community^13^. Specifically, sponges may release DOM that is more recalcitrant in nature^16^, but also includes certain labile metabolites (e.g., nucleosides, vitamins, amino acids – Fiore et al^14^.) that are readily incorporated by coral holobionts. The extent to which the composition of DM varies across sponge species is not yet well-characterized, but if it does vary, then this would have implications for species-specific efficiency of the trophic link between sponges and corals. In parallel, the physiological differences across coral species highlighted in our experimental work here also indicate a species-specific incorporation of sponge-derived nutrients by the holobiont. There may be additional recycling through the corals in our experiment, particularly for carbon, yet the assimilation of sponge-derived DM by corals of supports a complementary dimension to the original ‘sponge loop’. Our work indicates that in contrast to the ‘vicious circle’ and similar hypotheses on the potential negative interactions of sponges, macroalgae, and coral^9,72^, the trophic relationships between sponge species and others on the reef is complex with a range of types of interactions. The reciprocal exchange of nutrients between sponges and corals reveals that, at minimum, sponges are not necessarily counterproductive to coral survival on tropical reefs. In fact, the sponge to coral trophic link described here suggests that an ecosystem-based approach to coral restoration, particularly the inclusion of a sponge-community in close proximity to corals or a dietary supplement of sponge-derived exudates, may enhance coral growth, which has implications for improving coral aquaculture and management approaches as the coral species used in this study are currently commonly associated with reef restoration efforts.

## Methods

### Sponge and coral sample collection and maintenance

To encompass a characteristic sponge community for the Florida Keys reefs, we collected 8-10 individuals of each of six sponge species (*Niphates digitalis*, *Verongula rigida*, *Aplysina fulva*, *Aplysina cauliformis*, *Iotrochota birotulata*, *Callyspongia aculeata*; Supplementary Table 1) from Wonderland Reef (24.558694, -81.503528) within the Florida Keys National Marine Sanctuary (FKNMS) under a FL saltwater fishing permit (Permit #: I—H1R76333834 held by A.M. Reigel) and a FKNMS permit (FKNMS-2020–149) to work within the Sanctuary. These represent some of the most abundant sponge species in the Caribbean^73^ and they include a range in abundance and composition of microbial and algal symbiont communities^74^. Sponge collections were completed by scuba divers. Small individuals of *N. digitalis* and *C. aculeata* were collected whole; knives were used to sample large subsections (~12–15 cm diameter) from each individual sponge of the remaining four species. Upon collection, sponges were placed in Ziploc bags filled with seawater at depth. Sponges were kept in seawater-filled bags and immediately transported to the outdoor land-based nursery at Mote Marine Laboratory at Summerland Key, FL where they were transferred directly into a shaded, temperature- and pH-maintained flow-through tank (~1.9 L min^-1^) and allowed to acclimate for ~24 h. During transfer, sponges were not exposed to air and all efforts were made to touch them as little as possible. During the acclimation period, sponges were not disturbed.

For this experiment we used three corals with different morphologies including a branching hard coral, *Acropora cervicornis*, a boulder hard coral, *Orbicella faveolata*, and an octocoral, *Eunicea flexuosa*. *E. flexuosa* samples were also collected from Wonderland Reef under FL saltwater fishing permit (Permit #: I—H1R76333834 held by A.M. Reigel). Axial branch tips (hereby referred to as fragments) were clipped from each of 10 healthy *E. flexuosa* colonies located at depths of ~5–8 m. Fragments were kept in sea water and immediately transported to Mote Marine Laboratory where they were also placed in a shaded flow-through tank (~1.9 L min^-1^) to acclimate for ~24 h. The two hard coral species, *A. cervicornis* and *O. faveolata* were provided by Mote Marine Laboratory’s field- (*A. cervicornis*) and land-based (*O. faveolata*) nurseries as authorized under permits FKNMS-2015-163, FKNMS-2017–136, FKNMS-2021–171, and FKNMS-2021–172. All hard coral fragments were placed in the same flow-through tanks as the *E. flexuosa* samples and allowed to acclimate for ~24 h. Corals and sponges were not in the same tanks during the acclimation period.

### Stable isotope pulse-chase incubations

Following 24 h of acclimation time, whole sponges (*n* = 2 per species) and coral fragments (*n* = 4–5 per species) were removed from their respective acclimation tanks and immediately placed into labeled, sterile WhirlPak© bags and stored in the −20 °C freezer (i.e., T_0_ samples). The remaining sponges (*n* = 6-8 per species; Supplementary Table 1) were moved from the acclimation tank to a ‘pulse’ tank (94 L capacity) for a 3-hr incubation (Supplementary Fig. S1). A submersible aquarium pump was used to maintain water circulation and gas exchange in the ‘pulse’ tank, but it was not connected to the flow-through system. The ‘pulse’ tank was pre-filled with 84 liters of 0.22 µm filtered sea water spiked with isotopically labeled inorganic compounds in the following final concentrations: 0.1 g L^-1^ sodium bicarbonate (NaH^13^CO_3_), 0.01 g L^-1^ sodium nitrate (Na^15^NO_3_), and 0.01 g L^-1^ ammonium chloride (^15^NH_4_Cl). To mimic typical ratios of carbon and nitrogen concentrations on reefs, the sodium bicarbonate was 10x more concentrated than both inorganic nitrogen compounds. At the end of the 3 hr ‘pulse,’ a subset of sponges (‘pulse’ samples) was destructively sampled following the same procedure detailed above. The remaining enriched sponges (*n* = 5 per species) were then rinsed in the tank with flow-through seawater (~1.9 L min^-1^) for a 1 h ‘rinse’ period to flush away any labeled compounds that were not incorporated into the sponges. At the end of the rinse, two fragments from each coral species were placed into each of eight experimental tanks, five enriched sponge-containing tanks and three no-sponge control tanks, for the 6-hr ‘chase’ (Supplementary Fig. S1). All individuals were spaced throughout the tanks such that none were in physical contact. In the sponge-containing ‘chase’ tanks, sponges were located on one side of the tank and coral fragments on the opposite side to minimize the potential for exchange of particulate detritus from sponge to coral. A mesh separator screen was not used due to the potential for leachate to impact downstream dissolved organic carbon (DOC) analyses. During the duration of the ‘chase’ the flow-through system was turned off and a submersible aquarium pump was added to each tank to maintain circulation and gas exchange. Detritus production was expected to be low compared to the pumping activity of the sponges (e.g., ~0.005–0.3 L s^-1^ L^-1^ sponge^11^) and the estimated DOC output (e.g., ~90–100 µM^8^) in sponge exhalent water. While detritus production from cell turnover can be high in sponges, it is variable across species ranging from minimal to high detritus production^75^. No visible detritus production by the sponges was observed in the ‘pulse’ or ‘chase’ tanks, however, to reduce disturbance of detritus that may be produced, pumps in the ‘chase’ tanks were placed just under the surface of the water. A single fragment from each coral species was destructively sampled from each experimental tank twice during the ‘chase’: at the half point (T_3_) and at the end (T_6_). All enriched sponges were also destructively sampled at the end of the ‘chase.’ Following removal from the ‘pulse-chase’ experimental tanks, the coral fragments and sponges were immediately placed in individually labeled, sterile WhirlPak© bags and transferred to a −20 °C freezer.

To prepare for stable isotope analysis and Symbiodiniaceae cell counts, the coral fractions, host and Symbiodiniaceae, were manually separated by centrifuging. We did not genotype the dinoflagellates recovered in this study, so the terms ‘symbiotic micro-algae’ and ‘Symbiodiniaceae’ are used interchangeably to refer to any species of dinoflagellate that separated out during this process. Full methods for the separation process are detailed in the Supplementary Information, but briefly, scleractinian fragments were airbrushed with filtered sea water and the resulting tissue homogenate was centrifuged at high speed to pellet the Symbiodiniaceae cells while the host tissue remained suspended in the homogenate. For the octocoral, *E. flexuosa*, a similar process was followed, but rather than airbrushing, the entire fragment was lyopholized, ground into a powder, resuspended in MilliQ H_2_0, homogenized and then centrifugation was used to form a Symbiodiniaceae pellet and a host homogenate.

Following fraction separations, 50 ml of the Symbiodiniaceae fraction from each sample was transferred to a cryovial with 50 ml of 10% paraformaldehyde (PFA) to fix the cells for Symbiodiniaceae density estimates. Fixed Symbiodiniaceae samples were stored at 4 °C. The pure host homogenates and remaining Symbiodiniaceae pellets were stored at −20 °C.

### Processing of enriched water samples

During the ‘pulse-chase’ experiment, six 500 ml enriched water samples (*n* = 3) from the ‘pulse’ and (*n* = 3) from a subset of the sponge-containing aquaria ~1.5 h into the (‘chase’) were collected in acid-washed polycarbonate bottles for bulk dissolved organic carbon (DOC) and total nitrogen (TN) analysis. Detailed methods on the processing of enriched water samples can be found in the Supplementary Information.

### Stable isotope analysis

All sponge and Symbiodiniaceae tissues, and the octocoral host homogenate, were lyophilized, weighed, and the dried material was homogenized with a mortar and pestle. Homogenized samples were decalcified via exposure to 12 M HCL in desiccator chambers. Separate mortar and pestle sets and desiccator chambers were used for control and enriched samples. Subsamples of ~1–2.5 mg of decalcified tissue from each sample were packed into silver cups (Costech Analytical Technologies, Valencia, CA, USA). Scleractinian coral homogenates, made as previously detailed, contained filtered sea water, and if lyophilized, the salts could contaminate the host tissue. To remove the salts, host homogenates were passed through a sterile pre-weighed 1.6 mm GF/F filter that captured host material while sea water passed through and was discarded. The filters containing host material were frozen, dried overnight in a drying oven, weighed, decalcified as detailed above, and sent to the Marine Biological Laboratory (MBL) Stable Isotope Laboratory where they were also packed into silver cups.

Packed samples were analyzed at the MBL Stable Isotope Laboratory for *δ*^15^N and *δ*^13^C using a Europa 20-20 continuous-flow isotope ratio mass spectrometer interfaced with a Europa ANCA-SL elemental analyzer. The analytical precision based on replicate analyses of isotopically homogeneous international standards is +/- 0.1‰ for both *δ* ^15^N and *δ* ^13^C measurements, and about 1% relative to %N and %C measurements. Isotope values were initially expressed in delta notation (*δ* ^15^N and *δ* ^13^C), but to better elucidate the amount of ^13^C and ^15^N derived from sponges (i.e., micromoles of enriched C or N) that is incorporated into coral biomass, enrichment is also provided as incorporation and incorporation rate (i.e., enriched C or N in micromoles per hour) as documented in the Supplementary Information.

### Coral surface area measurements and symbiodiniaceae density

To obtain accurate Symbiodiniaceae abundance counts per coral fragment, we first calculated the fragment surface area following two well-documented methods: Image J measurements and the aluminum foil method. Details of each method can be found in the Supplementary Information. The surface area calculations from both methods were compared for all fragments to ensure a relative consensus between the methods. Results were largely similar between methods (Supplementary Fig. S2), but we chose to utilize the ImageJ method because although it resulted in slightly less conservative values it was more reproducible.

Symbiodiniaceae density (cells/cm^2^) was approximated for each coral fragment. Briefly, zooxanthellae cells were counted in a dilute subsample of the PFA-fixed Symbiodiniaceae homogenate (detailed above; cells/ml dilution), then extrapolated to obtain an estimated cell abundance for the entire fragment (cell/ml of host homogenate), and finally, total cell abundance was standardized to surface area of the fragment (cell/cm^2^) to obtain an estimated Symbiodiniaceae density. Detailed methods and full equations can be found in the Supplementary Information.

### Statistics and reproducibility

All statistical analyses were completed using functions from the R packages ‘vegan’^76^, and ‘STAT’^77^. Student’s *t*-tests were used to test for differences in data that met normality and/or homogeneity of variance assumptions including aquarium water DOC concentrations and intraspecies fraction-level differences in molar C:N ratios. Non-parametric tests, Kruskal-Wallis or permutational multivariate analysis of variance (PERMANOVA), were used to check for statistical differences on data that did not meet normality and/or homogeneity of variance assumptions including aquarium water TN, Symbiodiniaceae density, molar C:N ratios among holobiont species, and changes in δ values, incorporation, and incorporation rates of enriched isotopes in holobiont tissues. All PERMANOVA tests were run using the function *adonis2* in ‘vegan’ under a reduced model with 999 unique permutations. Non-parametric post-hoc tests for multivariate analyses were performed using the R package ‘pairwiseAdonis’^78^ with Bonferroni corrections. The detailed results of all PERMANOVA tests can be found in Supplementary Table S4, and all reported post-hoc pairwise tests can be found in the Supplementary Information (‘Supplementary Results’ section, pgs. 18–21).

### Reporting summary

Further information on research design is available in the Nature Portfolio Reporting Summary linked to this article.

### Supplementary information

Supplementary Information

Reporting Summary

## Data Availability

The raw enriched isotope and coral fragment (surface area and Symbiodiniaceae count) data generated and analyzed during the current study are available in the online repository of the Biological & Chemical Oceanography Data Management Office (BCO-DMO) at https://www.bco-dmo.org/dataset/889857^79^ and https://www.bco-dmo.org/dataset/880711^80^, respectively. All other relevant data supporting the findings of this study are available in the paper and its Supplementary Information file.
