# Supplementary material for: Sponge-derived matter is assimilated by coral holobionts
Source: Commun Biol. 2024 Feb 2;7:146. doi: 10.1038/s42003-024-05836-z (PMC10837432; doi:10.1038/s42003-024-05836-z)
Supplement: Supplementary file 1 — Supplementary Information [file 42003_2024_5836_MOESM1_ESM.pdf]

SUPPLEMENTARY INFORMATION

Sponge-derived matter is assimilated by coral holobionts

Authors: Reigel, Alicia M.[1], Easson, Cole G.[2], Apprill, Amy[3], Freeman, Christopher J.[4], Bartley, Michaela M.[5], Fiore, Cara L.[5]

Institutions:
[1]Washington and Lee University, Lexington, Virginia, USA
[2]Middle Tennessee State University, Murfreesboro, Tennessee, USA
[3]Woods Hole Oceanographic Institution, Woods Hole, Rhode Island, USA
[4]College of Charleston, Charleston, South Carolina, USA
[5]Appalachian State University, Boone, North Carolina, USA

Corresponding Author:
Name: Alicia M. Reigel
Affiliation: Biology Department, Washington and Lee University
Mailing Address: Howe Hall, Washington and Lee University, Lexington, VA 24450
Email: areigel@wlu.edu

Table of Contents

SUPPLEMENTARY METHODS *3*

Coral and Symbiodiniaceae Separations **3**

Processing of Enriched Water Samples **4**

Stable Isotope Analysis Equations **4**

Coral Surface Area Measurements **6**

Symbiodiniaceae Density **6**

SUPPLEMENTARY TABLES *8*

Supplementary Table S1. Total counts of sponge, coral and Symbiodiniaceae samples **8**

Supplementary Table S2. Mean $\delta^{13}C$ and $\delta^{15}N$ for sponge, coral, and Symbiodiniaceae tissues. **9**

Supplementary Table S3. Incorporation of ^{13}C & ^{15}N by coral and Symbiodiniaceae tissues. **10**

Supplementary Table S4. PERMANOVA Test Results **11**

SUPPLEMENTARY FIGURES *15*

Supplementary Figure S1. 'Pulse-chase' experimental setup visualization **15**

Supplementary Figure S2. Surface area (cm^2) of coral fragments **16**

Supplementary Figure S3. DOC & TN concentrations in 'pulse' seawater samples **17**

Supplementary Figure S4. Molar C:N ratio of non-enriched coral & Symbiodiniaceae tissues. **18**

Supplementary Figure S5. Symbiodiniaceae density in coral tissues. **19**

SUPPLEMENTARY RESULTS *20*

Pairwise PERMANOVA Test Results **20**

REFERENCES *24*

SUPPLEMENTARY METHODS

Coral and Symbiodiniaceae Separations

To prepare for downstream analyses, the coral fractions, host and symbiotic micro-algae (family Symbiodiniaceae), were manually separated. Symbiodiniaceae encompasses a diverse set of unicellular dinoflagellates[1] and corals are largely associated with those belonging to seven genera[2]. We did not genotype the dinoflagellates recovered in this study, so the terms 'symbiotic micro-algae' and 'Symbiodiniaceae' are used to refer to any species of dinoflagellate that separated out during this process. Scleractinian fragments were thawed and airbrushed with an aerosolized jet of 0.22 μm filtered seawater to physically separate the coral tissue and skeleton and suspend the coral tissue material into a homogenate. To separate host tissue from Symbiodiniaceae cells, the homogenate was centrifuged at 2000 x g for 3-5 minutes. Centrifugation formed a pellet comprised of Symbiodiniaceae cells and a homogenate of host material. The host homogenate was pipetted into a separate sterile 50 ml Falcon tube. The homogenate, Symbiodiniaceae pellet, and skeletal fragments were frozen and transported to Appalachian State University where they were stored at -20 C until further processing. At Appalachian State, host homogenates and Symbiodiniaceae pellets were thawed and checked for purity. Impure fractions were recombined, homogenized with a tissue homogenizer (maximum speed for ~15 sec) to physically separate Symbiodiniaceae cells from host tissue, and centrifuged (3000 x g, 6 min.) to pellet the Symbiodiniaceae cells. Following centrifuging, fractions were checked for purity under the microscope, and the process was repeated until at least 80% purity was reached.

E. flexuosa fractions were separated using a different process. First, the frozen coral fragments were lyophilized (Labconco™ FreeZone™ Bulk Tray Dryer) for 22-24 hours, until they were completely dry. Following lyopholization, the axial skeleton was removed, and the tissue was ground up using a mortar and pestle. Separate mortar and pestle sets were used for control and enriched samples. The ground tissue was weighed and then rehydrated in 10 ml of MilliQ water in a sterile 15 ml Falcon tube. Very quickly following rehydration, the sclerites (skeletal fragments) sank to the bottom of the tube and the remaining host homogenate was pipetted into a new tube taking care not to transfer the sclerites. The host homogenate was homogenized further using a tissue homogenizer for ~15 sec at maximum speed and centrifuged at 4000 x g for 5 minutes to separate the fractions. The centrifugation step was repeated as necessary until the host homogenate and Symbiodiniaceae pellets were at least 80% pure.

Following fraction separations, 50 ml of the Symbiodiniaceae fraction from each sample was transferred to a cryovial with 50 ml of 10% paraformaldehyde (PFA) to fix the cells for Symbiodiniaceae density estimates. Fixed Symbiodiniaceae samples were stored in the 4°C refrigerator. The pure host homogenates and remaining Symbiodiniaceae pellets were stored in the -20°C freezer.

Processing of Enriched Water Samples

During the 'pulse-chase' experiment, six 500 ml enriched water samples (n=3 from the 'pulse' and n=3 from a subset of the sponge-containing aquaria ~1.5 hrs into the 'chase') were collected in acid-washed polycarbonate bottles for bulk dissolved organic carbon (DOC) and total nitrogen (TN) analysis. Note that the tank water used for the 'pulse' was 0.22 µm filtered and the labeled bicarbonate provided is inorganic, thus, the DOC was composed of both background from the seawater and sponge-derived DOC. For the 'chase' samples, the water in the aquaria was not pre-filtered, so DOC sources include tank seawater, sponge- and coral-derived DOC, and possibly a small contribution from microbial production. TN includes both organic and inorganic nitrogen compounds, and for the 'pulse' was composed of background nitrogen, the labeled inorganic compounds, and sponge-derived nitrogenous dissolved matter (NDM). For the 'chase' the labeled compounds are replaced with possible microbe- and coral-produced NDM, though we expect this contribution was minimal.

Each enriched water sample was individually passed through a polytetrafluoroethylene (PTFE) 0.22 µm, 47 mm filter (Omnipore, EMD Millipore Corporation, Billerica, MA, USA) encased in an acid-washed in-line perfluoroalkoxy alkane (PFA) filter holder (Advantec, Cole-Parmer, Vernon Hills, IL, USA) using peristalsis (MasterFlex L/S pump and pump heads, Cole-Parmer, Vernon Hills, IL, USA). All filter tubing (Masterflex® L/S® Precision Pump Tubing, PharMed® BPT, L/S 25, Radnor, PA, USA) was acid-washed. Filtering rates were kept slow to avoid the bursting of microbial cells on the filter membrane which could cause sample contamination. Subsamples of the filtrate (25 ml) were collected in acid-washed, combusted 40ml amber EPA vials and acidified to pH 3 with 12M trace metal grade hydrochloric acid (HCL, OptimaTM, Fisher Chemical, Fisher Scientific, Hampton, NH, USA). EPA vials were wrapped in tinfoil to limit light exposure and stored at 4 ºC. The acidified samples were sent to Woods Hole Oceanographic Institute for dissolved organic carbon (DOC) and total dissolved nitrogen (TN) analysis using a Shimadzu TOC-V_{CSH}TOC analyzer[3].

Stable Isotope Analysis Equations

Carbon and nitrogen stable isotope ratios are expressed in standard delta notation and calculated using the equation from Fry[4] as follows:

$$\delta^{13}C \text{ or } \delta^{15}\mathrm{N} = \left[\left(\frac{R_{\text{sample}}}{(R_{\text{standard}}-1)}\right) * \ 1000\right],$$

where R_{sample} is the ratio of $^{13}C/^{12}C$ or $^{15}N/^{14}N$ in the sample material (sponge, coral, or Symbiodiniaceae), and R_{standard} is the known ratio of the isotopes in a standard reference material (Vienna Pee Dee Belemnite (VPDB) for C; R_{standard} = 0.011180, and for N; R_{standard}= 0.0036765). The final multiplication by 1000 is meant to amplify any small differences between samples and reference values. Values for δ are reported in permil units ($^{0}/_{00}$), and high δ values indicate enrichment of the heavy isotope while negative δ value indicate a sample depleted in heavy isotopes relative to the standard material.

Then, following the process outlined in Fry[4], we used the δ values to calculate fractional abundance (*F*; i.e., fractions of total C and N), which is preferred over δ for downstream calculations of enriched samples as it generates less errors. We start by rearranging the definition of δ as:

$$R_{\text{sample}} = \left[\left(\frac{\delta}{1000}\right) + 1\right] * R_{\text{standard}},$$

where, $R_{standard}$ is the same as previously noted for C and N,

Next, we use R_{sample} to calculate fractional abundance (F_{sample}) of ^{13}C or ^{15}N as follows:

$$F_{\text{sample}} = \frac{\delta + 1000}{(\delta + 1000) + \left(\frac{1000}{R_{\text{standard}}}\right)}$$

where δ is the $\delta^{13}C$ or $\delta^{15}N$ for the sample and the corresponding C or N $R_{standard}$ is used.

We then use F to calculate excess fractional abundance for an enriched sample (E_{sample}) as the difference between the fractional abundance of the enriched sample (F_{sample}) and the average fractional abundance of the background samples (F_{bkgd}), where background samples are all T_0 (i.e., initial) samples for each species and fraction.

$$E_{sample} = F_{sample} - F_{bkgd}.$$

Next, we used slightly modified versions of the equations put forth in Rix et al.[5] to calculate total ^{13}C or ^{15}N incorporation and incorporation rate. To calculate total incorporation (I) we multiplied the excess fractional abundance (E_{sample}) by the total C or N content (µmol) of the sample (A_{sample}):

$$I = E_{\text{sample}} * A_{\text{sample}}.$$

Lastly, we normalized the ^{13}C or ^{15}N incorporation (I) of each sample to its total C or N content (A_{sample}) and the number of hours the sample was exposed to the isotope (T) during the 'chase' (3 or 6 hrs) to obtain a biomass-specific incorporation rate of heavy isotope (IR_{Tracer}) into the tissue of each sample.

$$IR_{Tracer} = \frac{I}{(A_{\text{sample}} * T)}.$$

IR_{Tracer} is reported in the text results, Fig. 3, and Supplementary Table S4 as 'Incorporation Rate (µmol of ^{13}C or ^{15}N mmol of C or $N_{\text{coral or Symbiodiniaceae}}^{-1}$ hr^{-1}).' Values of $\delta^{13}C$, $\delta^{15}N$, total carbon and total nitrogen content (µmol of C or N) used in the above calculation are publicly available at https://www.bco-dmo.org/dataset/889857[6].

Coral Surface Area Measurements

Surface area was calculated for coral fragments following two well-documented methods: Image J measurements and the aluminum foil method[7]. ImageJ coral surface area measurements were completed utilizing planar photography and ImageJ software[8]. For *A. cervicornis* and *O.*

faveolata, fragment surface area was measured using the frozen, airbrushed skeleton for each sample, while for *E. flexuosa*, the entire fragment, prior to lypopholization as detailed above, was used. To obtain images of *A. cervicornis* and *E. flexuosa*, the fragments were held at an upright position, similar to their natural growth direction, and photographed from four sides (rotated 90°). *O. faveolata* are dome-shaped mounding corals, so photographs were only taken from above. A ruler was held in alignment with the fragments for scaling purposes. Photographs were individually uploaded to ImageJ and pixel dimensions were set using the straight-line tool and 'set scale' option. Using the polygon tool to drag an outline around the perimeter of the fragment, the enclosed area was calculated with the 'measure' function (in cm^2). The area of all four sides was summed to estimate the surface area of the skeletal fragment.

The aluminum foil method was completed based on published methods[7], but briefly, small pieces of aluminum foil were cut and carefully measured (cm^2) and weighed (g) to obtain a standard weight per unit of area (g/cm^2) for the foil. The foil used in this study had a standard weight per unit of area of 0.00618 g/cm^2. Coral fragments were carefully covered with aluminum foil and all excess foil was trimmed until there was no overlap. Each foil wrapping was carefully removed from the fragment, and weighed. The fragment surface area was calculated using the standard weight per unit area of the aluminum foil (Surface area of coral fragment = mass of coral fragment foil*0.006180525794 g/cm^2). All surface measurement data is publicly available at https://www.bco-dmo.org/dataset/880711[9].

Symbiodiniaceae Density

Symbiodiniaceae density ($cells/cm^2$) was estimated for each coral fragment using the following procedure. First, 20 µl of the PFA-fixed Symbiodiniaceae cells from each sample were stained with either 2 µl (*E. flexuosa*) or 5 µl (*A. cervicornis* and *O. faveolata*) of Trypan Blue to increase their visibility under the microscope. If the cells were too numerous to be counted accurately, they were further diluted with 175 µl of MilliQ water. A subsample (10 µl) of the dilution was then placed into the cell counting chamber of a hemocytometer (Marienfeld Superior Neubauer Improved Chamber) and cells were counted in 4 quadrants following the standard Neubauer protocol as suggested by Electron Microscopy Sciences (https://www.emsdiasum.com/microscopy/technical/datasheet/68052-14.aspx). Cell counts from all quadrants were summed. Symbiodiniaceae abundance in the dilution (cells/ml dilution) for each fragment was obtained by multiplying the total cell count by the dilution factor and the standard cell abundance for the hemocytometer (10,000 cells/ml per manufacturer) and dividing by the number of quadrants counted (4).

$$\boldsymbol{Dilution\ Abundance\ (cells/ml\ \ dilution)} = \frac{\boldsymbol{cell\ count * Dilution\ Factor * 10,000}}{\boldsymbol{4}}$$

Total Symbiodiniaceae abundance (cells/ml) per fragment was estimated by standardizing the dilution abundance to the total volume of its host homogenate (ml).

$$\boldsymbol{Symbiodiniaceae\ \ Abundance\ (cells/ml)} = Dilution\ Abundance * Volume\ host\ homogenate$$

Finally, the Symbiodiniaceae density for each fragment was calculated by standardizing the Symbiodiniaceae abundance to the total surface area of the fragment (cm^2) as determined by the ImageJ method detailed above.

$$\boldsymbol{Symbiodiniaceae\ Density\ (cell/cm^2)} = Symbiodiniaceae\ Abundance * fragment\ surface\ area\ (cm^2)$$

All data associated with the calculation of Symbiodiniaceae densities in the coral fragments is publicly available at https://www.bco-dmo.org/dataset/880711[9].

SUPPLEMENTARY TABLES

Supplementary Table S1. Total counts of sponge, coral and Symbiodiniaceae samples included at each point during the ‘pulse-chase’ experiment. Black cells indicate that no samples were collected at that time point. Note: There were a total of 8 experimental tanks (3 control, 5 enriched), but several coral and Symbiodiniaceae samples with abnormal ^{13}C or ^{15}N values (high or low depending on expected values for each treatment) were removed in an abundance of caution and this is reflected in the ‘chase’ sample counts.

**Species**	**Total Experimental Samples**	**Initial Control Samples (T=0)**	**‘pulse’ Samples**	**3-hr ‘chase’ Samples (T_3)**		**6-hr ‘chase’ Samples (T_6)**	
				**CONTROL**	**ENRICHED**	**CONTROL**	**ENRICHED**
A. cauliformis	10	2	3				5
A. fulva	9	2	2				5
V. rigida	9	2	2				5
N. digitalis	8	2	1				5
C. aculeata	8	2	1				5
I. birotulata	9	2	2				5
A. cervicornis	19	5		2	5	2	5
***A. cervicornis* Symbiodiniaceae**	19	5		2	5	2	5
O. faveolata	19	4		3	5	2	5
***O. faveolata* Symbiodiniaceae**	19	4		3	5	2	5
E. flexuosa	18	5		2	4	2	5
***E. flexuosa* Symbiodiniaceae**	19	5		3	5	2	4

Supplementary Table S2. Mean (± SD) $\delta^{13}C$ and $\delta^{15}N$ for sponge, coral, and Symbiodiniaceae tissues throughout the 6-hr 'pulse-chase' experiment. 'Pulse' column values are from sponge samples collected at the end of the 3-hr 'pulse.' T_0, indicates the initial samples taken prior to the start of the experiment, while T_3, and T_6 denote the two sampling times at the mid-point and the end of the 'chase,' respectively. Labeled isotopes provided to sponges via dissolved organic bicarbonate (^{13}C), ammonia and nitrate (^{15}N). Bolded red text in the "Change in $\delta^{13}C$" and "Change in $\delta^{15}N$" columns denotes a net loss of the heavy isotope during the 'chase,' while bolded blue text denotes a net gain of the heavy isotope during the 'chase.' Note that releases and gains are not necessarily significant. 'ND' is used to denote an experimental time point where samples were not collected for the indicated group. Sample groups without a standard deviation did not contain more than one individual.

**Sponges**	**$\delta^{13}C$ (‰)**					**$\delta^{15}N$ (‰)**				
	**T_0**	**'Pulse'**	**T_3**	**T_6**	**Change in $\delta^{13}C$ (T_6 - 'Pulse')**	**T_0**	**'Pulse'**	**T_3**	**T_6**	**Change in $\delta^{15}N$ (T_6 - 'Pulse')**
A. cauliformis	-18.6 ± 0.1	10.1 ± 22.3	ND	0.1 ± 7.1	**-10**	3.2 ± 0.8	661 ± 119	ND	546 ± 186	**-115**
A. fulva	-18.3 ± 0.3	1.6 ± 3.9	ND	3 ± 6.0	**1.4**	4.3 ± 1.6	473 ± 31.1	ND	442 ± 119	**-31**
V. rigida	-17.6 ± 0.1	0.2 ± 6.9	ND	-2.5 ± 4.8	**-2.6**	4.8 ± 0.4	1274 ± 520	ND	442 ± 80.1	**-832**
N. digitalis	-17.2 ±0.2	-17.0	ND	-16.6 ± 1.6	**0.4**	5.1 ± 0.4	988	ND	137 ± 36	**-851**
C. aculeata	-17.4 ± 0.3	-16.8	ND	-17.4 ± 0.2	**-0.6**	4.1 ± 0.5	1049	ND	121 ± 31.9	**-928**
I. birotulata	-17.6 ± 0.3	-17.4 ± 0.1	ND	-17.9 ± 0.6	**-0.5**	5.7 ± 0.6	570 ± 14.8	ND	371 ± 130	**-199**
**Coral Holobiont**	**T_0**	**'Pulse'**	**T_3**	**T_6**	**Change in $\delta^{13}C$ ($T_6 - T_0$)**	**T_0**	**'Pulse'**	**T_3**	**T_6**	**Change in $\delta^{15}N$ ($T_6 - T_0$)**
A. cervicornis	-18.5 ± 0.3	ND	-16.6 ± 1.1	-16.7 ± 1.3	**1.8**	4.2 ± 0.7	ND	154 ± 45	273 ± 54.2	**268.8**
O. faveolata	-25.2 ± 4.5	ND	-26.7 ± 0.5	-26.2 ± 1.9	**-1**	5.1 ± 0.7	ND	110 ± 41.5	165 ± 31.2	**160**
E. flexuosa	-19.4 ± 0.4	ND	-18.6 ± 0.6	-18 ± 0.6	**1.4**	6.4 ± 1.2	ND	126 ± 68.5	123 ± 77.9	**129.4**
***A. cervicornis* Symbiodiniaceae**	-18.4 ± 0.4	ND	-14.8 ± 2	-14.4 ± 2.6	**4**	3 ± 0.3	ND	756 ± 229	1829 ± 468	**1826.1**
***O. faveolata* Symbiodiniaceae**	-26.9 ± 1.1	ND	-22.7 ± 3.6	-23.7 ± 4.1	**3.2**	2 ± 1.2	ND	102 ± 35.3	181 ± 31.2	**183**
***E. flexuosa* Symbiodiniaceae**	-17.8 ± 0.3	ND	-16.8 ± 0.5	-15.9 ± 0.3	**1.9**	6.1 ± 1.6	ND	80.6 ± 40.2	104 ± 61.2	**97.9**

Supplementary Table S3. Total incorporation (µmol) and incorporation rates (heavy isotope (µmol)/total isotope in tissue (mmol) per hour) of ^{13}C and ^{15}N in sponge-derived dissolved matter by coral and Symbiodiniaceae during the 6-hr 'chase.' Data is given for both sampling time points (T_3 and T_6). Bolded blue text in T_6 columns denotes a value that is higher than its T_3 counterpart, conversely, bolded red text denotes a T_6 value that is lower than its T_3 counterpart. For total incorporation a blue value at T_6 would suggest a net gain of the heavy isotope in the tissue, and a red value a loss of the heavy isotope, between hours 3-6 of the 'chase.' For incorporation rate, a red value in T_6 indicates that the heavy isotope was assimilated into the coral or Symbiodiniaceae tissue faster in the first half of the 'chase,' while a blue value indicates a faster rate in the last half of the 'chase.' A bolded black value in either column indicates no difference across 'chase' time points. Note differences are not necessarily significant.

**Species and Fraction**	**Carbon (^{13}C) Incorporation**			
	**T_3**		**T_6**	
	**Incorporation (µmol)**	**Incorporation Rate (µmol ^{13}C mmol $C_{coral\ or\ Sym}^{-1}$ hr^{-1})**	**Incorporation (µmol)**	**Incorporation Rate (µmol ^{13}C mmol $C_{coral\ or\ Sym}^{-1}$ hr^{-1})**
A. cervicornis	0.0021	0.0069	**0.0019**	**0.0032**
O. faveolata	-0.0008	-0.0056	**-0.0004**	**-0.0018**
E. flexuosa	0.0002	0.0032	**0.0003**	**0.0027**
***A. cervicornis* Symbiodiniaceae**	0.0026	0.0132	**0.0029**	**0.0072**
***O. faveolata* Symbiodiniaceae**	0.0025	0.0154	**0.0019**	**0.0058**
***E. flexuosa* Symbiodiniaceae**	0.0002	0.0035	**0.0003**	0.0035

**Species and Fraction**	**Nitrogen (^{15}N) Incorporation**			
	**T_3**		**T_6**	
	**Incorporation (µmol)**	**Incorporation Rate (µmol ^{15}N mmol N $N_{coral\ or\ Sym}^{-1}$ hr^{-1})**	**Incorporation (µmol)**	**Incorporation Rate (µmol ^{15}N mmol N $N_{coral\ or\ Sym}^{-1}$ hr^{-1})**
A. cervicornis	0.0066	0.1828	**0.0119**	**0.1633**
O. faveolata	0.0023	0.1281	**0.0028**	**0.0973**
E. flexuosa	0.0012	0.1454	**0.0013**	**0.0711**
***A. cervicornis* Symbiodiniaceae**	0.0288	0.9139	**0.0778**	**1.104**
***O. faveolata* Symbiodiniaceae**	0.0039	0.1267	**0.0065**	**0.1115**
***E. flexuosa* Symbiodiniaceae**	0.0009	0.0907	**0.0007**	**0.0746**

Supplementary Table S4. PERMANOVA results of multivariate tests reported in the text. Significance was obtained from p-values calculated by permutations of the residuals under a reduced model using 999 unique permutations. Associated post-hoc pairwise PERMANOVAS were completed with Bonferroni corrections and are detailed in the Supplementary Results (pgs. 18-21). Asterisks denote significant differences (p<0.05).

Factor(s)	df	SS	R^2	Pseudo-F	P(perm)
**a. δ ^{13}C for T_0 & enriched coral & Symbiodiniaceae from sampling points T_3 & T_6**					
Species	2	1234.20	0.7373	161.9887	0.001 *
Fraction	1	60.05	0.0389	15.7642	0.001 *
Time	2	61.76	0.0369	8.1055	0.004 *
Species:Fraction	2	0.91	0.0005	0.1191	0.884
Species:Time	4	12.89	0.0077	0.8460	0.517
Fraction:Time	2	25.06	0.0150	3.2892	0.045 *
Time:Fraction:Species	4	20.14	0.0120	1.3216	0.293
Residual	68	259.05	0.1547		
Total	85	1674.06			
**b. δ ^{13}C for control coral & Symbiodiniaceae tissues from 'chase' sampling points T_0, T_3 & T_6**					
Species	2	896.05	0.9720	1314.4441	0.001 *
Fraction	1	5.95	0.0065	17.4657	0.001 *
Species:Fraction	2	3.81	0.0041	5.5848	0.011 *
Residual	47	16.02	0.0174		
Total	52	921.83			
**c. δ ^{13}C for control *A. cervicornis* coral tissues from 'chase' sampling points T_0, T_3 & T_6**					
Time	2	0.3657	0.4175	2.1503	0.184
Residual	6	0.5100	0.5825		
Total	8	0.8756			
**d. δ ^{13}C for control *A. cervicornis* Symbiodiniaceae tissues from 'chase' sampling points T_0, T_3 & T_6**					
Time	2	0.3706	0.3961	1.9676	0.278
Residual	6	0.5650	0.6039		
Total	8	0.9656			
**e. δ ^{13}C for control *O. faveolata* coral tissues from 'chase' sampling points T_0, T_3 & T_6**					
Time	2	0.1304	0.1028	0.2864	0.789
Residual	5	1.1383	0.8972		
Total	7	1.2688			
**f. δ ^{13}C for control *O. faveolata* Symbiodiniaceae tissues from 'chase' sampling points T_0, T_3 & T_6**					
Time	2	0.1097	0.0215	0.0659	
Residual	6	4.9925	0.9785		
Total	8	5.1022			
**g. δ ^{13}C for control *E. flexuosa* coral tissues from 'chase' sampling points T_0, T_3 & T_6**					
Time	2	2.8039	0.4877	2.8563	
Residual	6	2.9450	0.5123		
Total	8	5.7489			
**h. δ ^{13}C for control *E. flexuosa* Symbiodiniaceae tissues from 'chase' sampling points T_0, T_3 & T_6**					
Time	2	0.3519	0.1685	0.6078	0.593
Residual	6	1.7370	0.8315		
Total	8	2.0889			
**i. ^{13}C incorporation (umol) of enriched coral & Symbiodiniaceae fractions**					
Fraction	1	2.3e-05	0.1360	8.8165	0.007 *
Residual	56	1.5e-04	0.8640		
Total	57	1.7e-04			
**j. ^{13}C incorporation (umol) of enriched coral tissues only**					
Species	2	3.4329e-05	0.6984	27.5052	0.001 *

Supplementary Table S4 continued on pg. 11

Supplementary Table S4 continued from pg. 10

Factor(s)	df	SS	R^2	Pseudo-F	P(perm)
Time	1	6.8000e-08	0.0014	0.1091	0.756
Species:Time	1	4.0400e-07	0.0082	0.3239	0.738
Residual	23	1.4353e-05	0.2920		
Total	28	4.9155e-05			
**k. ^{13}C incorporation (umol) of enriched Symbiodiniaceae tissues only**					
Host Species	2	3.3058e-05	0.3427	6.1103	0.011 *
Time	1	9.0000e-08	0.0009	0.0334	0.859
Host Species:Time	2	1.0910e-06	0.01131	0.2016	0.828
Residual	23	6.2217e-05	0.64503		
Total	28	9.6456e-05			
**l. ^{13}C incorporation rate (µmol of ^{13}C mmol of $C_{coral\ or\ Symbiodiniaceae}^{-1}$ hr^{-1}) of enriched coral and Symbiodiniaceae tissues**					
Fraction	1	0.0007	0.2141	15.255	0.001 *
Residual	56	0.0025	0.7860		
Total	57	0.0032			
**m. ^{13}C incorporation rate (µmol of ^{13}C mmol of C_{coral}^{-1} hr^{-1}) of enriched coral tissues from 'chase' exposure intervals T_0-T_3 & T_0-T_6**					
Species	2	0.0004	0.6421	29.7461	0.001 *
Time Interval	1	0.0000	0.0001	0.0118	0.908
Species:Time Interval	2	0.0001	0.1096	5.0754	0.021 *
Residual	23	0.0002	0.2482		
Total	28	0.0007			
**n. ^{13}C incorporation rate (µmol of ^{13}C mmol of $C_{Symbiodiniaceae}^{-1}$ hr^{-1}) of enriched Symbiodiniaceae tissues from 'chase' exposure intervals T_0-T_3 & T_0-T_6**					
Host Species	2	0.0003	0.1610	2.7675	0.072
Time Interval	1	0.0002	0.1121	3.8563	0.061
Host Species:Time Interval	2	0.0001	0.0581	0.9981	0.396
Residual	23	0.0012	0.6689		
Total	28	0.0018			
**o. δ ^{15}N for T_0 & enriched coral & Symbiodiniaceae from sampling points T_3 & T_6**					
Species	2	3414557	0.19753	98.057	0.001 *
Fraction	1	1280488	0.07407	73.544	0.001 *
Time	2	3048922	0.17638	87.557	0.001 *
Species:Fraction	2	2537777	0.14681	72.878	0.001 *
Species:Time	4	2731825	0.15803	39.225	0.001 *
Fraction:Time	2	1098499	0.06355	31.546	0.001 *
Time:Fraction:Species	4	1990549	0.11515	28.581	0.001 *
Residual	68	1183958	0.06849		
Total	85	17286575			
**p. δ ^{15}N for control coral & Symbiodiniaceae tissues from 'chase' sampling points T_0, T_3 & T_6**					
Species	2	162.3	0.0248	0.6727	0.597
Fraction	1	421.0	0.0644	3.4894	0.049 *
Species:Fraction	2	287.5	0.04395	1.1914	0.348
Residual	47	5670.8	0.2669		
Total	52	6541.6			
**q. δ ^{15}N for control *A. cervicornis* coral tissues from 'chase' sampling points T_0, T_3 & T_6**					
Time	2	9.0036	0.7646	9.7441	0.011 *
Residual	6	2.7720	0.2354		
Total	8	11.7756			
**r. δ ^{15}N for control *E. flexuosa* coral tissues from 'chase' sampling points T_0, T_3 & T_6**					
Time	2	644.30	0.5838	4.2073	0.047 *
Residual	6	459.41	0.4162		
Total	8	1103.72			

Supplementary Table S4 continued on pg. 12

Supplementary Table S4 continued from pg. 11

Factor(s)	df	SS	R^2	Pseudo-F	P(perm)
**s. δ ^{15}N for <u>control</u> *O. faveolata* coral tissues from 'chase' sampling points T_0, T_3 & T_6**					
Time	2	0.1304	0.1028	0.2864	0.789
Residual	5	1.1383	0.8972		
Total	7	1.2688			
**t. δ ^{15}N for <u>control</u> *A. cervicornis* Symbiodiniaceae tissues from 'chase' sampling points T_0, T_3 & T_6**					
Time	2	26.088	0.7454	8.7819	0.057
Residual	6	8.912	0.2546		
Total	8	35.00			
**u. δ ^{15}N for <u>control</u> *E. flexuosa* Symbiodiniaceae tissues from 'chase' sampling points T_0, T_3 & T_6**					
Time	2	4.0036	0.2372	0.9331	0.435
Residual	6	12.8720	0.4763		
Total	8	16.8756			
**v. δ ^{15}N for <u>control</u> *O. faveolata* Symbiodiniaceae tissues from 'chase' sampling points T_0, T_3 & T_6**					
Time	2	417.72	0.3190	4.4054	0.263
Residual	6	891.68	0.6810		
Total	8	1309.40			
**w. ^{15}N incorporation (umol) of <u>enriched</u> coral & Symbiodiniaceae fractions**					
Fraction	1	0.8780	0.0734	4.4375	0.013 *
Residual	56	11.0800	0.9266		
Total	57	11.958			
**x. ^{15}N incorporation (umol) of <u>enriched</u> coral tissues only**					
Species	2	2.6303	0.6859	30.4456	0.001 *
Time	1	0.1039	0.0271	2.4063	0.105
Species:Time	1	0.1073	0.0280	1.2417	0.293
Residual	23	0.9935	0.2591		
Total	28	3.8351			
**y. ^{15}N incorporation (umol) of <u>enriched</u> Symbiodiniaceae tissues only**					
Host Species	2	5.5951	0.7722	66.9491	0.001 *
Time	1	0.1972	0.0272	4.7185	0.008 *
Host Species:Time	1	0.4919	0.0679	5.8862	0.001 *
Residual	23	0.9611	0.1327		
Total	28	7.2452			
**z. ^{15}N incorporation rate (µmol of ^{15}N mmol of $N_{coral\ or\ Symbiodiniaceae}^{-1}$ hr^{-1}) of <u>enriched</u> coral and Symbiodiniaceae tissues**					
Fraction	1	0.7732	0.1030	6.4299	0.008 *
Residual	56	6.7344	0.8970		
Total	57	7.5076			
**aa. ^{15}N incorporation rate (µmol of ^{15}N mmol of N_{coral}^{-1} hr^{-1}) of <u>enriched</u> coral tissues from 'chase' exposure intervals T_0-T_3 & T_0-T_6**					
Species	2	0.4382	0.2799	5.6072	0.006 *
Time Interval	1	0.1255	0.0802	3.2124	0.061
Species:Time Interval	2	0.1031	0.0659	1.3193	0.254
Residual	23	0.8986	0.5741		
Total	28	1.5654			
**bb. ^{15}N incorporation rate (µmol of ^{15}N mmol of $N_{Symbiodiniaceae}^{-1}$ hr^{-1}) of <u>enriched</u> Symbiodiniaceae tissues from 'chase' exposure intervals T_0-T_3 & T_0-T_6**					
Host Species	2	4.1261	0.7982	50.1907	0.001 *
Time Interval	1	0.0446	0.0086	1.0849	0.315
Host Species:Time Interval	2	0.0530	0.0103	0.6443	0.580
Residual	23	0.9454	0.1829		
Total	28	5.1691			

Supplementary Table S4 continued on pg. 13

Supplementary Table S4 continued from pg. 12					
**Factor(s)**	**df**	**SS**	**R^2**	**Pseudo-F**	**P(perm)**
**cc. Molar C:N Ratio for T_0 coral & Symbiodiniaceae tissues only**					
Species	2	0.0206	0.2127	8.2689	0.003 *
Fraction	1	0.0101	0.1038	0.0667	0.016 *
Species:Fraction	2	0.0401	0.4134	16.0717	0.001 *
Residual	21	0.0262	0.2701		
Total	26	0.0971			
**dd. Symbiodiniaceae density (cells/cm^2) in all coral fragments**					
Host Species	2	9.8206	0.7744	131.4812	0.001 *
Treatment	1	0.0838	0.0066	2.2443	0.133
Time	2	0.2999	0.0237	4.0157	0.012 *
Host Species:Treatment	2	0.2072	0.0163	2.7742	0.051
Host Species: Time	4	0.4592	0.0362	3.0743	0.010 *
Treatment:Time	1	0.0652	0.0051	1.7449	0.198
Host Species:Treatment:Time	2	0.1026	0.0081	1.3738	0.265
Residual	44	1.6432	0.1296		
Total	58	12.6818			
**ee. Symbiodiniaceae density (cells/cm^2) in *A. cervicornis* coral fragments**					
Time	2	0.0315	0.0691	0.631	0.576
Residual	17	0.4238	0.9309		
Total	19	0.4553			
**ff. Symbiodiniaceae density (cells/cm^2) in *O. faveolata* coral fragments**					
Time	2	0.0975	0.2065	2.2115	0.108
Residual	17	0.3748	0.7935		
Total	19	0.4723			
**gg. Symbiodiniaceae density (cells/cm^2) in *E. flexuosa* coral fragments**					
Time	2	0.7517	0.3887	5.0877	0.006 *
Residual	16	1.1820	0.6113		
Total	18	1.9337			

SUPPLEMENTARY FIGURES

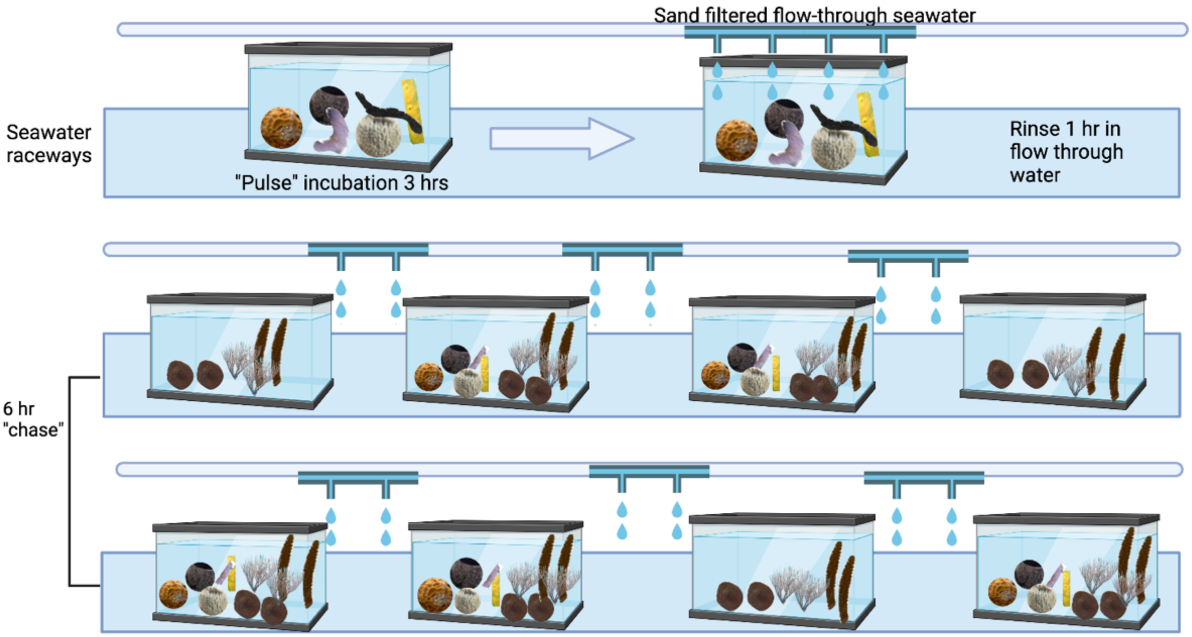

Supplementary Figure S1. 'Pulse-chase' experimental setup visualization. Following a 24-hr acclimation period, individuals of six species of emergent Caribbean sponges (n=6-8 each) were placed in a single aquarium with dissolved isotopically-labeled inorganic nutrients (bicarbonate containing ^{13}C, and nitrate and ammonium both containing ^{15}N) for a 3-hr 'pulse' (top left). Following the pulse, five enriched sponges from each species were moved to a new sand-filtered flow-through seawater tank for a 1-hr rinse (top right) and remaining individuals (n=1-3) were destructively sampled, hereby known as 'pulse' sponge samples. Following the rinse, enriched sponges were separated into 5 randomly assigned 20-gal tanks contained in two larger mesocosms without flow-through, for the 6-hr 'chase' (Bottom). Each 'chase' tank also contained fragments of three coral species (*Acropora cervicornis*, *Orbicella faveolata*, *Eunicea flexuosa*, n=2 per species), none of which were initially enriched. The 'chase' setup resulted in five experimental tanks containing corals and enriched sponges, and three control tanks containing only corals. At 3-hrs into the 'chase' (T_3), a single fragment of each coral species was destructively sampled from each tank. At the end of the 6-hr 'chase' (T_6) all remaining corals and sponges (n=1 per species per tank) were destructively sampled. All organisms were spaced throughout the tanks such that none were in physical contact and in 'chase' tanks sponges were located on one side of the tank and corals on the other side to minimize the potential for exchange of particulate detritus from sponge to coral. Organisms and tanks in image are not drawn to scale. Visualization was made using BioRender.

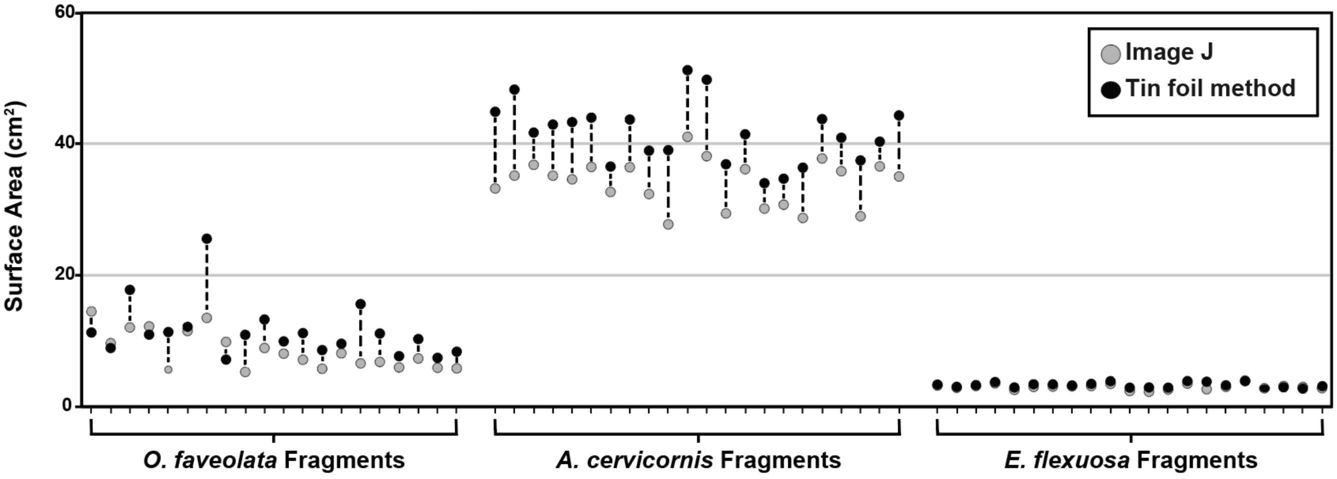

Supplementary Figure S2. Surface area (cm^2) comparison of coral fragments using two different measurement methods: Image J (gray circles) and Tin Foil Method (black circles). Fragments are grouped by coral species, *Orbicella faveolata* (n=20), *Acropora cervicornis* (n=22) and *Eunicea flexuosa* (n=20), and each dashed line within the species group connects the circles corresponding to one individual fragment that has been measured with both methods. For *E. flexuosa* individuals, dashed lines are not visible due to low disparity in surface area measurement between methods.

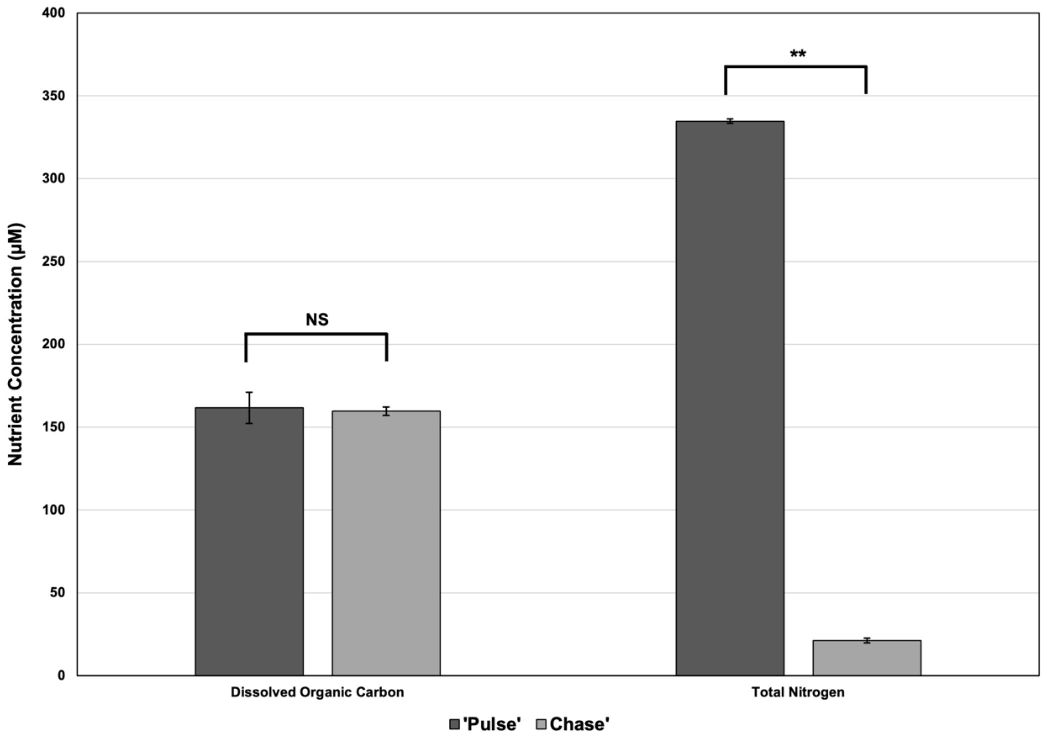

Supplementary Figure S3. Concentration (µM ± SD) of dissolved organic matter (DOC) and total nitrogen in the aquaria water samples from the end of the 'pulse' (dark gray; n=3) and a subset of the tanks containing coral fragments during the 'chase' (light gray; n=3). NS denotes no significant difference in DOC concentration between the two time points, while two asterisks denote a significant difference ($p<0.0001$) in total nitrogen between the two time points.

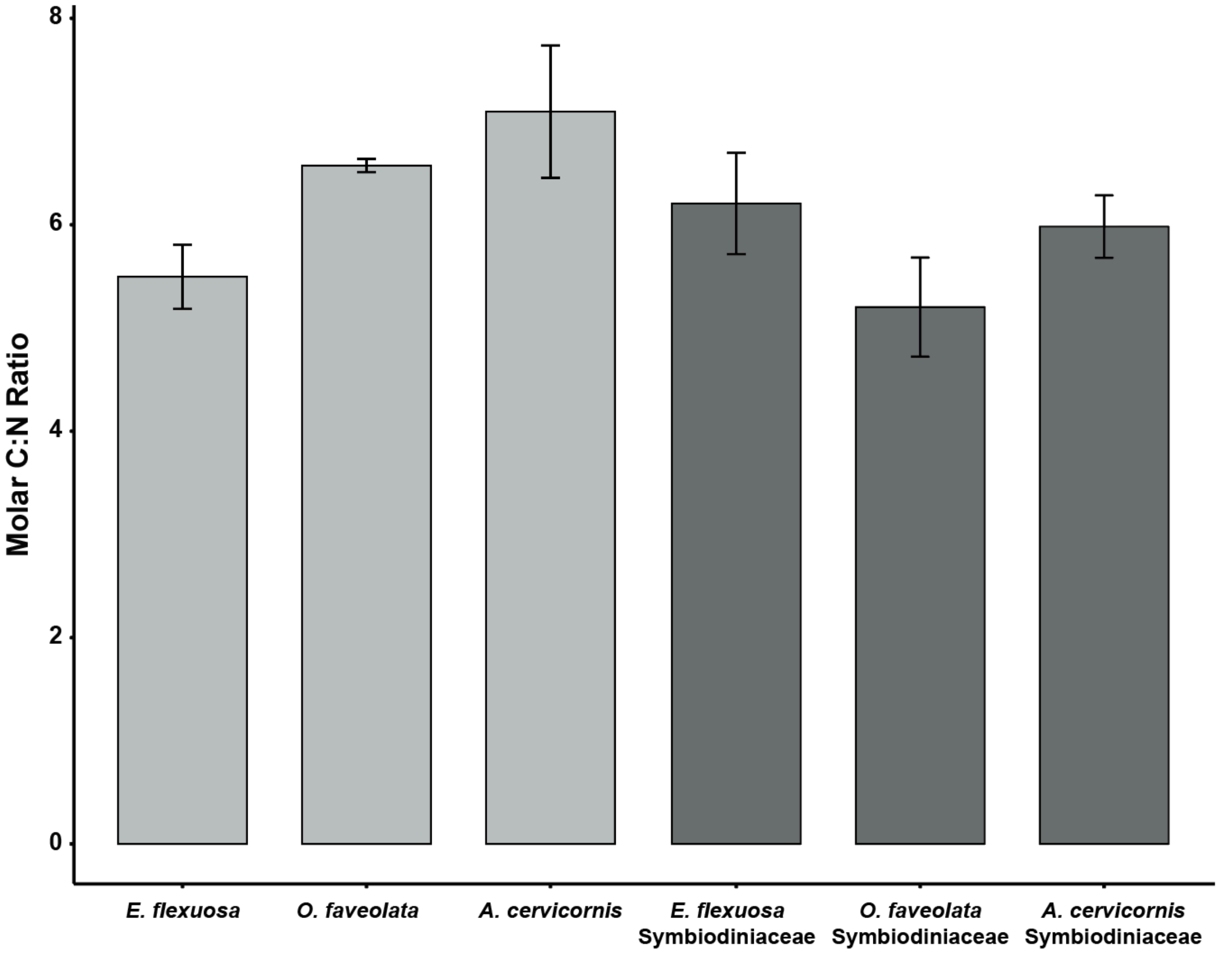

Supplementary Figure S4. Molar C:N ratio (± SD) of coral (light gray) and Symbiodiniaceae tissue (dark gray) for all non-enriched T_0 (i.e., initial) samples.

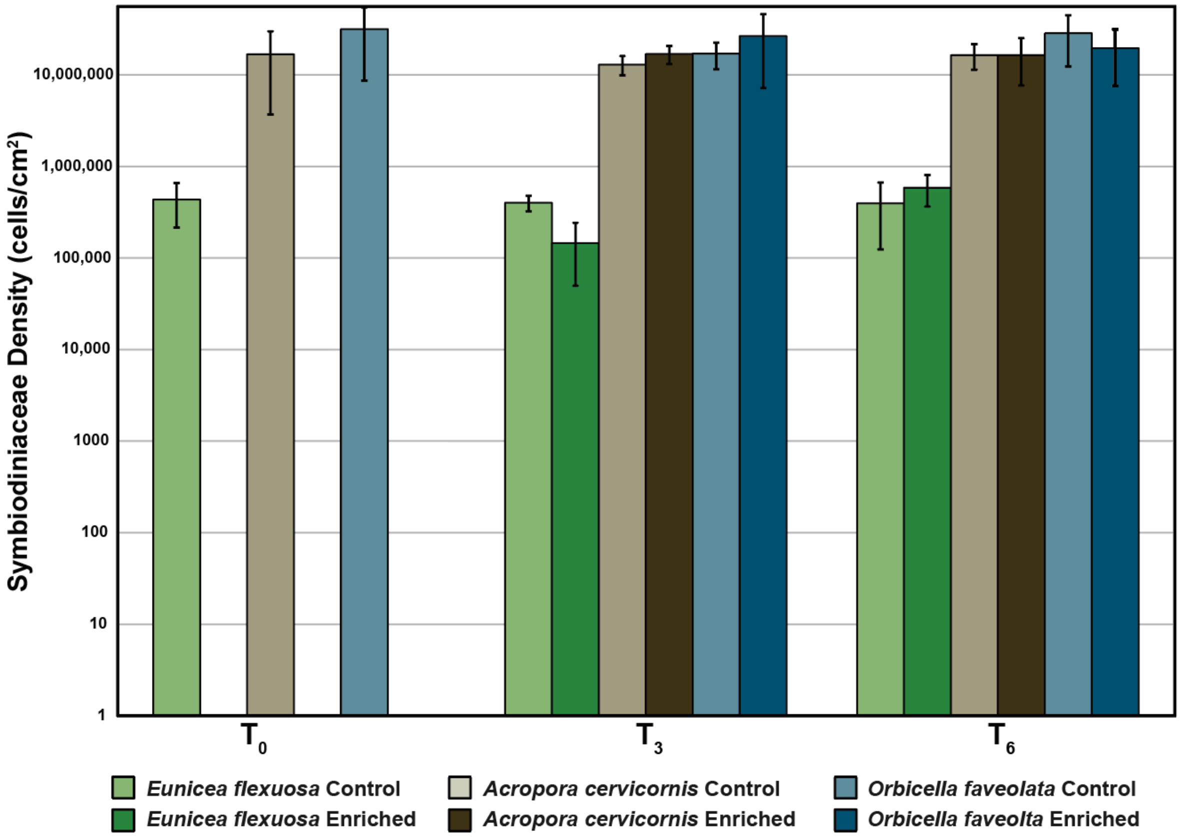

Supplementary Figure S5. Symbiodiniaceae density (cells/cm^2 ± SD) in tissues from *Eunicea flexuosa* (brown) *Acropora cervicornis* (blue), and *Orbicella faveolata* (yellow). The lighter shaded bar for each coral species indicates Symbiodiniaceae abundance in control samples and the darker shaded bar denotes abundance in enriched samples (i.e., exposed to labeled isotopes via sponge-derived DM during the 'chase'). Abundance is shown for three time-points including initial, 3-hrs, and 6-hrs into the 'chase' (T_0, T_3, and T_6, respectively). Note that Symbiodiniaceae density is shown on a log^{10} scale.

SUPPLEMENTARY RESULTS

Pairwise PERMANOVA Test Results

Here we report the results from the post-hoc pairwise tests associated with the PERMANOVA tests reported in Supplementary Table S4. All post-hoc tests were completed using the '*pairwiseadonis*' package in R with 999 permutations and Bonferroni corrections. Only pairwise results that are reported in text are included here. For access to unreported pairwise tests please contact the corresponding author. Asterisks denote significant ($p<0.05$) differences.

Pairwise tests for Supplementary Table S4j – ^{13}C Incorporation for Enriched Coral Tissue

**Ofav_vs_Eflex**	Df	SumOfSqs	R2	F	Pr(>F)
Species	1	3.4655e-06	0.60753	27.8247	0.002 *
Time	1	3.0800e-07	0.05399	2.4728	0.149
Species:Time	1	6.2500e-08	0.01096	0.5020	0.470
Residual	15	1.8682e-06	0.32751		
Total	18	5.7043e-06	1.00000		

**Ofav_vs_Acer**	Df	SumOfSqs	R2	F	Pr(>F)
Species	1	3.3169e-05	0.69305	37.2230	0.001 *
Time	1	3.3000e-08	0.00069	0.0368	0.847
Species:Time	1	4.0000e-07	0.00836	0.4490	0.478
Residual	16	1.4257e-05	0.29790		
Total	19	4.7859e-05	1.00000		

**Eflex_vs_Acer**	Df	SumOfSqs	R2	F	Pr(>F)
Species	1	1.4018e-05	0.52422	16.7129	0.001 *
Time	1	9.2000e-09	0.00034	0.0110	0.914
Species:Time	1	1.3220e-07	0.00494	0.1576	0.686
Residual	15	1.2581e-05	0.47049		
Total	18	2.6740e-05	1.00000		

Pairwise tests for Supplementary Table S4m – ^{13}C Incorporation Rates for Enriched Coral Tissue

Ofav_vs_Eflex	Df	SumOfSqs	R2	F	Pr(>F)
Species	1	0.00020664	0.64368	40.1740	0.001 *
Time	1	0.00001506	0.04691	2.9280	0.121
Species:Time	1	0.00002217	0.06907	4.3107	0.055
Residual	15	0.00007715	0.24034		
Total	18	0.00032102	1.00000		

Ofav_vs_Acer	Df	SumOfSqs	R2	F	Pr(>F)
Species	1	0.00038609	0.64350	43.0957	0.001 *
Time	1	0.00000003	0.00004	0.0030	0.962
Species:Time	1	0.00007053	0.11755	7.8722	0.010 *
Residual	16	0.00014334	0.23891		
Total	19	0.00059999	1.00000		

Eflex_vs_Acer	Df	SumOfSqs	R2	F	Pr(>F)
Species	1	2.2565e-05	0.14232	3.3348	0.082
Time	1	2.2664e-05	0.14295	3.3494	0.089
Species:Time	1	1.1823e-05	0.07457	1.7473	0.219
Residual	15	1.0150e-04	0.64016		
Total	18	1.5855e-04	1.00000		

Pairwise tests for Supplementary Table S4o – δ ^{15}N for T_0 & Enriched Coral & Symbiodiniaceae Tissue

**Ofav_vs_Eflex**	Df	SumOfSqs	R2	F	Pr(>F)
Species	1	11580	0.03964	6.7291	0.012 *
HolobiontFraction	1	1505	0.00515	0.8744	0.365
Time	2	187377	0.64145	54.4397	0.001 *
Species:HolobiontFraction	1	1348	0.00462	0.7835	0.388
Species:Time	2	11277	0.03861	3.2765	0.046 *
HolobiontFraction:Time	2	1900	0.00650	0.5519	0.558
Species:HolobiontFraction:Time	2	1405	0.00481	0.4083	0.680
Residual	44	75722	0.25922		
Total	55	292115	1.00000		

**Ofav_vs_Acer**	Df	SumOfSqs	R2	F	Pr(>F)
Species	1	2352041	0.14613	95.924	0.001 *
HolobiontFraction	1	2009917	0.12487	81.972	0.001 *
Time	2	3802555	0.23625	77.541	0.001 *
Species:HolobiontFraction	1	1866721	0.11598	76.132	0.001 *
Species:Time	2	1849750	0.11492	37.720	0.001 *
HolobiontFraction:Time	2	1665632	0.10348	33.965	0.001 *
Species:HolobiontFraction:Time	2	1421029	0.08829	28.977	0.001 *
Residual	46	1127906	0.07008		
Total	57	16095551	1.00000		

**Eflex_vs_Acer**	Df	SumOfSqs	R2	F	Pr(>F)
Species	1	2699741	0.16442	106.664	0.001 *
HolobiontFraction	1	1894026	0.11535	74.831	0.001 *
Time	2	3532477	0.21514	69.783	0.001 *
Species:HolobiontFraction	1	1861432	0.11337	73.544	0.001 *
Species:Time	2	2179639	0.13275	43.058	0.001 *
HolobiontFraction:Time	2	1569993	0.09562	31.015	0.001 *
Species:HolobiontFraction:Time	2	1518138	0.09246	29.990	0.001 *
Residual	46	1164288	0.07091		
Total	57	16419733	1.00000		

Pairwise tests for Supplementary Table S4q – δ ^{15}N for Control *A. cervicornis* Coral Tissue

**T=0 vs T=3**	Df	SumOfSqs	R2	F	Pr(>F)
Time	1	7.8223	0.76135	15.951	0.041 *
Residual	5	2.4520	0.23865		
Total	6	10.2743	1.00000		

**T=0 vs T=6**	Df	SumOfSqs	R2	F	Pr(>F)
Time	1	3.388	0.58014	6.9086	0.104
Residual	5	2.452	0.41986		
Total	6	5.840	1.00000		

--

**T=3 vs T=6**	Df	SumOfSqs	R2	F	Pr(>F)
Time	1	0.64	0.5	2	0.6667
Residual	2	0.64	0.5		
Total	3	1.28	1.0		

Pairwise tests for Supplementary Table S4r – δ ^{15}N for Control *E. flexuosa* Coral Tissue

**T=0 vs T=3**	Df	SumOfSqs	R2	F	Pr(>F)
Time	1	586.96	0.56122	6.3951	0.041 *
Residual	5	458.91	0.43878		
Total	6	1045.87	1.00000		

**T=0 vs T=6**	Df	SumOfSqs	R2	F	Pr(>F)
Time	1	0.112	0.01718	0.0874	0.824
Residual	5	6.408	0.98282		
Total	6	6.520	1.00000		

**T=3 vs T=6**	Df	SumOfSqs	R2	F	Pr(>F)
Time	1	422.30	0.48219	1.8624	0.3333
Residual	2	453.51	0.51781		
Total	3	875.81	1.00000		

Pairwise tests for Supplementary Table S4x – ^{15}N Incorporation for Enriched Coral Tissue

**Ofav_vs_Eflex**	Df	SumOfSqs	R2	F	Pr(>F)
Species	1	0.42585	0.33646	7.7705	0.010 *
Time	1	0.01499	0.01185	0.2736	0.697
Species:Time	1	0.00280	0.00221	0.0511	0.944
Residual	15	0.82205	0.64949		
Total	18	1.26569	1.00000		

**Ofav_vs_Acer**	Df	SumOfSqs	R2	F	Pr(>F)
Species	1	1.25985	0.61634	34.9720	0.001 *
Time	1	0.13249	0.06482	3.6778	0.049 *
Species:Time	1	0.07536	0.03687	2.0919	0.136
Residual	16	0.57639	0.28198		
Total	19	2.04410	1.00000		

**Eflex_vs_Acer**	Df	SumOfSqs	R2	F	Pr(>F)
Species	1	2.26269	0.74233	57.6610	0.001 *
Time	1	0.11532	0.03783	2.9387	0.088
Species:Time	1	0.08147	0.02673	2.0762	0.161
Residual	15	0.58862	0.19311		
Total	18	3.04809	1.00000		

Pairwise tests for Supplementary Table S4y – ^{15}N Incorporation for Enriched Symbiodiniaceae Tissue

**Acer_vs_Ofav**	Df	SumOfSqs	R2	F	Pr(>F)
Species	1	2.5761	0.72417	129.126	0.001 *
Time	1	0.2394	0.06731	12.002	0.002 *
Species:Time	1	0.4226	0.11879	21.181	0.001 *
Residual	16	0.3192	0.08973		
Total	19	3.5573	1.00000		

**Acer_vs_Eflex**	Df	SumOfSqs	R2	F	Pr(>F)
Species	1	3.6851	0.72937	66.6652	0.001 *
Time	1	0.3032	0.06001	5.4847	0.018 *
Species:Time	1	0.2350	0.04651	4.2509	0.028 *
Residual	15	0.8292	0.16411		
Total	18	5.0524	1.00000		

**Ofav_vs_Eflex**	Df	SumOfSqs	R2	F	Pr(>F)
Species	1	2.14304	0.69246	41.5435	0.001 *
Time	1	0.10806	0.03492	2.0948	0.157
Species:Time	1	0.06995	0.02260	1.3560	0.269
Residual	15	0.77378	0.25002		
Total	18	3.09484	1.00000		

Pairwise tests for Supplementary Table S4cc – Molar C:N Ratio for T_0 coral & Symbiodiniaceae Tissue

**Ofav_vs_Acer**	Df	SumOfSqs	R2	F	Pr(>F)
Species	1	0.015377	0.20786	11.5117	0.006 *
HolobiontFraction	1	0.039718	0.53687	29.7333	0.001 *
Species:HolobiontFraction	1	0.001520	0.02055	1.1379	0.296
Residual	13	0.017365	0.23473		
Total	16	0.073981	1.00000		

Ofav_vs_Eflex	Df	SumOfSqs	R2	F	Pr(>F)
Species	1	0.000281	0.00584	0.2406	0.644
HolobiontFraction	1	0.000647	0.01345	0.5542	0.467
Species:HolobiontFraction	1	0.032024	0.66524	27.4128	0.001 *
Residual	13	0.015187	0.31548		
Total	16	0.048139	1.00000		

Acer_vs_Eflex	Df	SumOfSqs	R2	F	Pr(>F)
Species	1	0.014560	0.23902	11.7203	0.007 *
HolobiontFraction	1	0.000870	0.01429	0.7006	0.423
Species:HolobiontFraction	1	0.025609	0.42040	20.6147	0.002 *
Residual	16	0.019876	0.32629		
Total	19	0.060916	1.00000		

Pairwise tests for Supplementary Table S4dd – Symbiodiniaceae density in all coral fragments

**Eflex_vs_Acer**	Df	SumOfSqs	R2	F	Pr(>F)
Species	1	7.4167	0.75637	114.87	0.001 *
Residual	37	2.3889	0.24363		
Total	38	9.8057	1.00000		

**Eflex_vs_Ofav**	Df	SumOfSqs	R2	F	Pr(>F)
Species	1	6.7213	0.7364	103.36	0.001 *
Residual	37	2.4059	0.2636		
Total	38	9.1273	1.0000		

**Acer_vs_Ofav**	Df	SumOfSqs	R2	F	Pr(>F)
Species	1	0.70074	0.43036	28.709	0.001 *
Residual	38	0.92754	0.56964		
Total	39	1.62828	1.00000		

Pairwise tests for Supplementary Table S4gg – Symbiodiniaceae density in *E. flexuosa* coral fragments

T0_vs_T3	Df	SumOfSqs	R2	F	Pr(>F)
Time	1	0.28165	0.20847	2.6338	0.083
Residual	10	1.06936	0.79153		
Total	11	1.35101	1.00000		

T0_vs_T6	Df	SumOfSqs	R2	F	Pr(>F)
Time	1	0.07615	0.16681	2.0021	0.144
Residual	10	0.38036	0.83319		
Total	11	0.45651	1.00000		

T3_vs_T6	Df	SumOfSqs	R2	F	Pr(>F)
Time	1	0.71347	0.43833	9.3648	0.001 ***
Residual	12	0.91424	0.56167		
Total	13	1.62770	1.00000		

REFERENCES

1. LaJeunesse, T. C. Zooxanthellae. *Curr. Biol.* **30**, R1110–R1113 (2020).

3. LaJeunesse, T. C. *et al.* Systematic revision of *Symbiodiniaceae* highlights the antiquity and diversity of coral endosymbionts. *Curr. Biol.* **28**, 2570-2580.e6 (2018).

1. Hansell, D. & Carlson, C. Marine Dissolved Organic Matter and the Carbon Cycle. *Oceanography* **14**, (2001).

4. Fry, B. *Stable isotope ecology*. (Springer, 2006). doi:10.1007/0-387-33745-8.

5. Rix, L. *et al.* Differential recycling of coral and algal dissolved organic matter via the sponge loop. *Funct. Ecol.* **31**, 778–789 (2017).

9. Reigel, A. M. *et al.* Isotopic analysis of ^{13}C and ^{15}N for sponges, coral, and zooxanthellae (family *Symbiodiniaceae*) used in a 'pulse-chase' experiment to examine the uptake of sponge-derived nutrients by the coral holobiont. (2023) doi:10.26008/1912/BCO-DMO.889857.1.

7. Marsh, J. A. Primary Productivity of Reef-Building Calcareous Red Algae. *Ecology* **51**, 255–263 (1970).

5. Schneider, C. A., Rasband, W. S. & Eliceiri, K. W. NIH image to ImageJ: 25 years of image analysis. *Nat. Methods* **9**, 671–675 (2012).

9. Reigel, A. M. *et al.* Coral fragment surface area calculations utilizing two methods (tin foil and Image J) and corresponding zooxanthellae count data. (2022) doi:10.26008/1912/BCO-DMO.880711.1.
