# Supplementary material for: Sponge-derived matter is assimilated by coral holobionts
Source: Commun Biol. 2024 Feb 2;7:146. doi: 10.1038/s42003-024-05836-z (PMC10837432; doi:10.1038/s42003-024-05836-z)
Supplement: Supplementary file 2 — Reporting Summary [file 42003_2024_5836_MOESM2_ESM.pdf]

nature portfolio

Corresponding author(s): Alicia M. Reigel

Last updated by author(s): 01/04/2024

Reporting Summary

Nature Portfolio wishes to improve the reproducibility of the work that we publish. This form provides structure for consistency and transparency in reporting. For further information on Nature Portfolio policies, see our Editorial Policies and the Editorial Policy Checklist.

Statistics

For all statistical analyses, confirm that the following items are present in the figure legend, table legend, main text, or Methods section.

n/a	Confirmed	
☐	☒	The exact sample size (*n*) for each experimental group/condition, given as a discrete number and unit of measurement
☐	☒	A statement on whether measurements were taken from distinct samples or whether the same sample was measured repeatedly
☐	☒	The statistical test(s) used AND whether they are one- or two-sided<br>*Only common tests should be described solely by name; describe more complex techniques in the Methods section.*
☐	☒	A description of all covariates tested
☐	☒	A description of any assumptions or corrections, such as tests of normality and adjustment for multiple comparisons
☐	☒	A full description of the statistical parameters including central tendency (e.g. means) or other basic estimates (e.g. regression coefficient) AND variation (e.g. standard deviation) or associated estimates of uncertainty (e.g. confidence intervals)
☐	☒	For null hypothesis testing, the test statistic (e.g. *F*, *t*, *r*) with confidence intervals, effect sizes, degrees of freedom and *P* value noted<br>*Give P values as exact values whenever suitable.*
☒	☐	For Bayesian analysis, information on the choice of priors and Markov chain Monte Carlo settings
☐	☒	For hierarchical and complex designs, identification of the appropriate level for tests and full reporting of outcomes
☒	☐	Estimates of effect sizes (e.g. Cohen's *d*, Pearson's *r*), indicating how they were calculated

Our web collection on statistics for biologists contains articles on many of the points above.

Software and code

Policy information about availability of computer code

Data collection	The software ANC NT System (v. 1.99d) was used to run the Europa 20-20 continuous-flow isotope ratio mass spectrometer interfaced with a Europa ANCA-SL elemental analyzer to measure stable istopes. The resulting data was processed using the software ANCREPRO. Further data processing, including drift, linearity corrections, and final normalizations were performed offline in Microsoft Excel.
Data analysis	R (version 4.2.2) was used for all statistical analyses and all R packages used are listed and cited (with associated parameters for each analysis) in the methods and/or supplemental information.

For manuscripts utilizing custom algorithms or software that are central to the research but not yet described in published literature, software must be made available to editors and reviewers. We strongly encourage code deposition in a community repository (e.g. GitHub). See the Nature Portfolio guidelines for submitting code & software for further information.

Data

Policy information about availability of data

All manuscripts must include a data availability statement. This statement should provide the following information, where applicable:

- Accession codes, unique identifiers, or web links for publicly available datasets
- A description of any restrictions on data availability
- For clinical datasets or third party data, please ensure that the statement adheres to our policy

The raw enriched isotope and coral fragment (surface area and Symbiodiniaceae count) data generated and analyzed during the current study are available in the

online repository of the Biological & Chemical Oceanography Data Management Office (BCO-DMO) at https://www.bco-dmo.org/dataset/889857 and https://www.bco-dmo.org/dataset/880711, respectively. All other relevant data supporting the findings of this study are available in the paper and its Supplementary Information file.

Human research participants

Policy information about studies involving human research participants and Sex and Gender in Research.

Reporting on sex and gender	*Use the terms sex (biological attribute) and gender (shaped by social and cultural circumstances) carefully in order to avoid confusing both terms. Indicate if findings apply to only one sex or gender; describe whether sex and gender were considered in study design whether sex and/or gender was determined based on self-reporting or assigned and methods used. Provide in the source data disaggregated sex and gender data where this information has been collected, and consent has been obtained for sharing of individual-level data; provide overall numbers in this Reporting Summary. Please state if this information has not been collected. Report sex- and gender-based analyses where performed, justify reasons for lack of sex- and gender-based analysis.*
Population characteristics	*Describe the covariate-relevant population characteristics of the human research participants (e.g. age, genotypic information, past and current diagnosis and treatment categories). If you filled out the behavioural & social sciences study design questions and have nothing to add here, write "See above."*
Recruitment	*Describe how participants were recruited. Outline any potential self-selection bias or other biases that may be present and how these are likely to impact results.*
Ethics oversight	*Identify the organization(s) that approved the study protocol.*

Note that full information on the approval of the study protocol must also be provided in the manuscript.

Field-specific reporting

Please select the one below that is the best fit for your research. If you are not sure, read the appropriate sections before making your selection.

☐ Life sciences ☐ Behavioural & social sciences ☒ Ecological, evolutionary & environmental sciences

For a reference copy of the document with all sections, see nature.com/documents/nr-reporting-summary-flat.pdf

Ecological, evolutionary & environmental sciences study design

All studies must disclose on these points even when the disclosure is negative.

Study description	A stable isotope 'pulse-chase' experiment was used to trace the uptake of sponge-derived matter by the coral holobiont. Caribbean sponges were incubated with inorganic 13C and 15N tracer compounds, then placed into aquaria containing fragments from three coral species (Acropora cervicornis, Orbicella faveolata, and Eunicea flexuosa). The holobiont host (i.e., coral tissue) and symbiotic micro-algae (Symbiodiniaceae) fractions were manually separated following the 'pulse-chase' experiment and changes in 13C and 15N in their tissues were compared across experimental time points, between fractions, and among coral species.
Research sample	To encompass a characteristic sponge community for the Florida Keys reefs, we collected 8-10 individuals of each of six sponge species (Niphates digitalis, Verongula rigida, Aplysina fulva, Aplysina cauliformis, Iotrochota birotulata, Callyspongia aculeata. We also used three corals with different morphologies including a branching hard coral, Acropora cervicornis, a boulder hard coral, Orbicella faveolata, and an octocoral, Eunicea flexuosa.
Sampling strategy	All sponge and coral samples from the 'pulse-chase' experiment were destructively sampled. Sponges (n=2 per species) and corals (n=4-5 fragments per species) were sampled prior to the start of the 'pulse-chase' experiment. The remaining sponges (n=6-8 per species) were moved to the 'pulse' tank for exposure to labeled-inorganic nutrients. Enriched sponges (n=1-3 per species) were sampled at the end of the 3-hr 'pulse.' The remaining enriched sponges were rinsed for 1-hr and then placed into five 'chase' tanks with coral fragments of all three species. There were also three control tanks that contained coral fragments, but no enriched sponges. Coral fragments (n=1 fragment per species per tank) from all eight 'chase' tanks were sampled 3-hrs into the 'chase' (T3) and at the end of the 6-hr 'chase' (T6) for a total of 5 enriched and 3 control fragments per coral species per time 'chase' time point.
Data collection	The experiment was setup and the data collection was completed by co-author Cole G. Easson and two student research assistants.
Timing and spatial scale	All sponge and Eunicea flexuosa samples were collected on 9-Dec-2020 from Wonderland Reef (24.558694, -81.503528) within the Florida Keys National Marine Sanctuary (FKNMS). Also on 9-Dec-2020, the Acropora cervicornis samples were obtained from the Mote Marine Laboratory field-based nursery (24.562747, -81.400455) and the Orbicella faveolata samples from their land-based nursery. All A. cervicornis and O. faveolata samples were obtained and used as authorized under permits FKNMS-2015-163, FKNMS-2017-136, FKNMS-2021-171, and FKNMS-2021-172.<br><br>The stable isotope 'pulse-chase' experiment was started and completed on 10-Dec-2020. The experiment ran for total of 10 hours (3-hr 'pulse', 1-hr rinse, 6-hr 'chase').

Data exclusions	No samples were excluded from stable isotope measurements, but three coral samples (Ef-iso-T3-7, Ef-iso-T6-2, Ac-iso-I-2) were not utilized for Symbiodiniaceae counts because either no Symbiodiniaceae cells were seen in the sample or, during the host and symbiont separations, the ethanol cleaning step was not fully rinsed, which causes the Symbiodiniaceae to burst and they are unable to be counted. Further, four control (i.e., non-enriched) coral samples (Of_T6_7, AC_T3_3, AC_T6_1, and EF_T6_1) and their corresponding zooxanthellae have $\delta^{15}N$ and $\delta^{13}C$ values that suggest they may have been contaminated by the labeled isotopes at some point during the sample preparation process. We included the raw data for these samples in the data deposited online (https://www.bco-dmo.org/dataset/889857), but they were excluded from all downstream analyses in this study.
Reproducibility	*Describe the measures taken to verify the reproducibility of experimental findings. For each experiment, note whether any attempts to repeat the experiment failed OR state that all attempts to repeat the experiment were successful.*
Randomization	Initial samples of both sponges and corals were randomly selected from the acclimation tanks. Control and enriched 'chase' tanks were randomly assigned, but all tanks were placed inside of the same larger mesocosm. All sponges and coral fragments in the 'pulse-chase' experiment were randomly selected for destructive sampling at the appropriate time points, but at the end of the experiment all remaining samples were used.
Blinding	Blinding was not possible during the 'pulse-chase' experiment nor the prepartion of the samples for stable isotope analyses due to the potential for contamination if enriched samples were not kept separate from non-enriched samples. Further, blinding was not necessary in the stable isotope analysis and that work was completed by a contract facility without any help or input from any authors.

Did the study involve field work? ☒ Yes ☐ No

Field work, collection and transport

Field conditions	Sample collection day: 9-Dec-2020: Seawater temperature at the collection site was normal for the season (~27C) and air temperature was cooler (~13C). Surface conditions were calm and there was little current. We did not measure turbidity, but per diver notes the Wonderland Reef site had very low visibility
Location	Florida Keys National Marine Sanctuary Sites: Wonderland Reef (24.558694, -81.503528) and Mote Marine Laboratory field-based nursery (24.562747, -81.400455).
Access & import/export	All research activities were approved under Florida Keys National Marine Sanctuary Permit FKNMS-2020-149. All sponge and E. flexuosa samples were collected as allowed under FL saltwater fishing permit (Permit #: I—H1R76333834 held by A.M. Reigel). All A. cervicornis and O. faveolata fragments obtained from Mote Marine Laboratory nurseries were acquired and used as authorized under permits FKNMS-2015-163, FKNMS-2017-136, FKNMS-2021-171, and FKNMS-2021-172.
Disturbance	E. flexuosa samples were obtained by clipping axial branch tips of larger colonies and long-term disturbance is negligible. Most sponge samples were collected from larger individuals that will repair leaving negligible long-term distrubance. When whole sponges were collected (e.g., for Niphates digitalis) they were removed from the benthos as gently as possible and with all efforts to leave surrounding habitat untouched.

Reporting for specific materials, systems and methods

We require information from authors about some types of materials, experimental systems and methods used in many studies. Here, indicate whether each material, system or method listed is relevant to your study. If you are not sure if a list item applies to your research, read the appropriate section before selecting a response.

Materials & experimental systems

n/a	Involved in the study	
☒	☐	Antibodies
☒	☐	Eukaryotic cell lines
☒	☐	Palaeontology and archaeology
☒	☐	Animals and other organisms
☒	☐	Clinical data
☒	☐	Dual use research of concern

Methods

n/a	Involved in the study	
☒	☐	ChIP-seq
☒	☐	Flow cytometry
☒	☐	MRI-based neuroimaging
